# Intergenic and Repeat Transcription in Human, Chimpanzee and Macaque Brains Measured by RNA-Seq

**DOI:** 10.1371/journal.pcbi.1000843

**Published:** 2010-07-01

**Authors:** Augix Guohua Xu, Liu He, Zhongshan Li, Ying Xu, Mingfeng Li, Xing Fu, Zheng Yan, Yuan Yuan, Corinna Menzel, Na Li, Mehmet Somel, Hao Hu, Wei Chen, Svante Pääbo, Philipp Khaitovich

**Affiliations:** 1 Partner Institute for Computational Biology, Shanghai, China; 2 Max-Planck-Institute for Evolutionary Anthropology, Leipzig, Germany; 3 Graduate School of the Chinese Academy of Sciences, Shanghai, China; 4 Max Planck Institute for Molecular Genetics, Berlin, Germany; 5 Max Delbrück Centrum für Molekulare Medizin, Berlin Institute for Medical Systems Biology, Berlin-Buch, Germany; Cornell University, United States of America

## Abstract

Transcription is the first step connecting genetic information with an organism's phenotype. While expression of annotated genes in the human brain has been characterized extensively, our knowledge about the scope and the conservation of transcripts located outside of the known genes' boundaries is limited. Here, we use high-throughput transcriptome sequencing (RNA-Seq) to characterize the total non-ribosomal transcriptome of human, chimpanzee, and rhesus macaque brain. In all species, only 20–28% of non-ribosomal transcripts correspond to annotated exons and 20–23% to introns. By contrast, transcripts originating within intronic and intergenic repetitive sequences constitute 40–48% of the total brain transcriptome. Notably, some repeat families show elevated transcription. In non-repetitive intergenic regions, we identify and characterize 1,093 distinct regions highly expressed in the human brain. These regions are conserved at the RNA expression level across primates studied and at the DNA sequence level across mammals. A large proportion of these transcripts (20%) represents 3′UTR extensions of known genes and may play roles in alternative microRNA-directed regulation. Finally, we show that while transcriptome divergence between species increases with evolutionary time, intergenic transcripts show more expression differences among species and exons show less. Our results show that many yet uncharacterized evolutionary conserved transcripts exist in the human brain. Some of these transcripts may play roles in transcriptional regulation and contribute to evolution of human-specific phenotypic traits.

## Author Summary

Phenotypic differences between closely related species, such as humans and chimpanzees, might be determined to a large extent by differences between their transcriptomes. Recent studies using microarray and high-throughput sequencing technologies have demonstrated that beside annotated genes, a large proportion of the human genome can be transcriptionally active. Little is known, however, about the extent and the conservation of human brain transcripts located outside of the known genes' boundaries. Here, we use high-throughput transcriptome sequencing to characterize the non-ribosomal transcriptome of the human cerebellum and compare it to the transcriptomes of chimpanzee and rhesus macaque. Our results show that close to 40% of all transcripts expressed in the human brain map within repetitive elements. By contrast, less then 10% of the human brain transcriptome corresponds to non-repetitive intergenic regions. Nonetheless, within these regions we identify more than a thousand novel highly transcribed evolutionary conserved locations. Some of the intergenic transcripts show distinct human-specific expression and may have contributed to evolution of human-specific phenotypic traits.

## Introduction

Transcriptome studies conducted by various methodologies, such as conventional sequencing, tiling arrays, and, most recently, high-throughput sequencing, have consistently indicated that a large proportion of transcription takes place outside known gene boundaries (see [1], [2] and references therein). Among human tissues, the brain transcriptome is one of the most complex [3], [4]. Changes in expression of brain transcripts have been suggested to play an essential role in evolution of the human phenotype [5]. Indeed, expression of protein-coding genes differs greatly between humans and one of our closest relatives [6], [7], [8]. Furthermore, comprehensive analysis of approximately 1% of the human and chimpanzee brain transcriptomes using tiling arrays found multiple instances of differential expression outside annotated gene regions [9].

To systematically characterize the transcriptome in a particular brain region, cerebellar cortex, and identify its human-specific features, we performed high-throughput sequencing using the Illumina platform to analyze transcripts expressed in ten humans, four chimpanzees, and five rhesus macaques. All individuals are adult males (Table S1). While most previous studies [1], [2], [4], [10] have focused on the RNA fraction carrying poly(A) tails, we sequenced all transcripts present in the total RNA, excluding ribosomal RNA (rRNA) and depleting RNA transcripts shorter than 200 nucleotides (nt). Our experimental strategy is similar to the strategy used to characterize total transcriptome of HeLa cells [11], [12], with the difference that these studies either focused on the 3′-region of the transcript or sequenced mixture of polyA+ and polyA− transcripts with predominant part of polyA-enriches ones. To reduce within-species variation, we pooled the total RNA from brains of four to five individuals of the same species into one sample. To estimate technical as well as remaining within-species biological variation, we sequenced two independent human samples, each comprising total RNA from five individuals.

## Results

### Brain transcriptome composition

**Figure 1:**
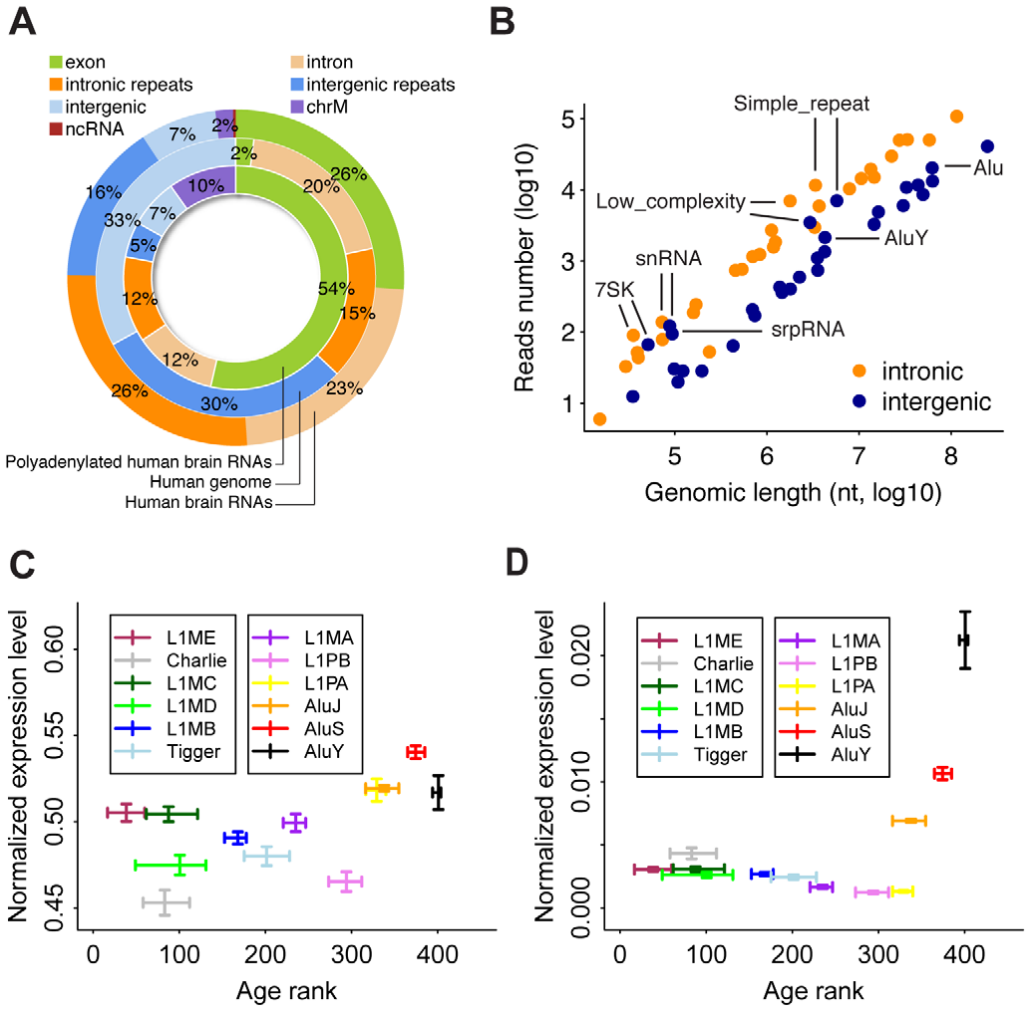
Composition of human brain transcriptome and transcription of repetitive elements. (A) Outer circle: average proportions of transcriptome sequence reads from the two human samples that map within annotated exons (green), introns (light orange), intronic repeats (orange), intergenic repeats (blue), intergenic regions (light blue), mitochondrial DNA (purple), and ncRNA (maroon). Middle circle: the proportions occupied by the corresponding regions in the human genome. Inner circle: the proportions of transcriptome sequence reads for polyadenylated human brain RNA (data adopted from [26]). (B) The transcriptional activity of repeat families located within introns (orange) or intergenic regions (blue) plotted against the total genomic length occupied by the family (see Materials and Methods for details). The labels indicate the repeat families with elevated expression levels. (C and D) The expression levels of twelve TE families normalized by the total genomic length of the corresponding family (C) and by the length corresponding to expressed repeats (D), plotted against their age rank. The expression level 95% confidence intervals are calculated by 1,000 bootstraps over sequence reads. The age rank and the corresponding confidence intervals are plotted according to [39]. Higher age rank corresponds to evolutionary younger TE families.

For each sample, we obtained an average of ∼10,000,000 sequence reads of 36 nt corresponding to ∼7,200,000 unique sequences. From these reads, we can map on average 51% to the corresponding reference genomes and annotated exon junctions (Table S2). Excluding the remaining sequences mapping to rRNA, we find that in humans 26% of the reads map to annotated exons and exon junctions, 2% - to mitochondrial genes, and less than 1% - to annotated non-coding RNA (ncRNA) (Figure 1A). Although these proportions are much greater than the corresponding genomic fractions (Figure 1A), they still represent less than a third of the total non-ribosomal human brain transcriptome. The remaining reads map within introns and intergenic regions (49% and 23% of the transcriptome, respectively). Such a distribution of transcriptome reads is not unique to humans, but shared among the three primate species studied (Figure S1).

### Repeat transcription

Within intronic and intergenic regions, more than half of transcription originates from repetitive sequence elements, occupying in total ∼42% of the entire transcriptome (Figure 1A). This proportion is substantially greater than that reported in the human brain using cap-selected transcript tags (∼10%) [13]. For most of the repeat families, the expression is proportional to the genome fraction occupied (Figure 1B). Still, for some, such as simple and low complexity repeats, as well as repeat families derived from functional ncRNA, such as snRNA, snpRNA and 7SK RNA, the expression level is higher than expected from the repeat family size alone in all three species studied (Figure S2).

More than 90% of repeats present in the human genome result from transposable element (TE) activity taking place over hundreds of millions of years. Estimating the transcriptional activity of different TE families, we find that the most recently expanded ones, the Alu elements, show elevated transcriptional activity per genomic fraction occupied by the family (Figure 1C). The effect is more obvious when normalizing by the genomic fraction occupied by repeat elements actually expressed in brain (Figure 1D). We find the same effect in the other two species (Figure S3), indicating that elevated expression of certain Alu elements in brain might be widespread among primates.

### Intergenic transcription

**Figure 2:**
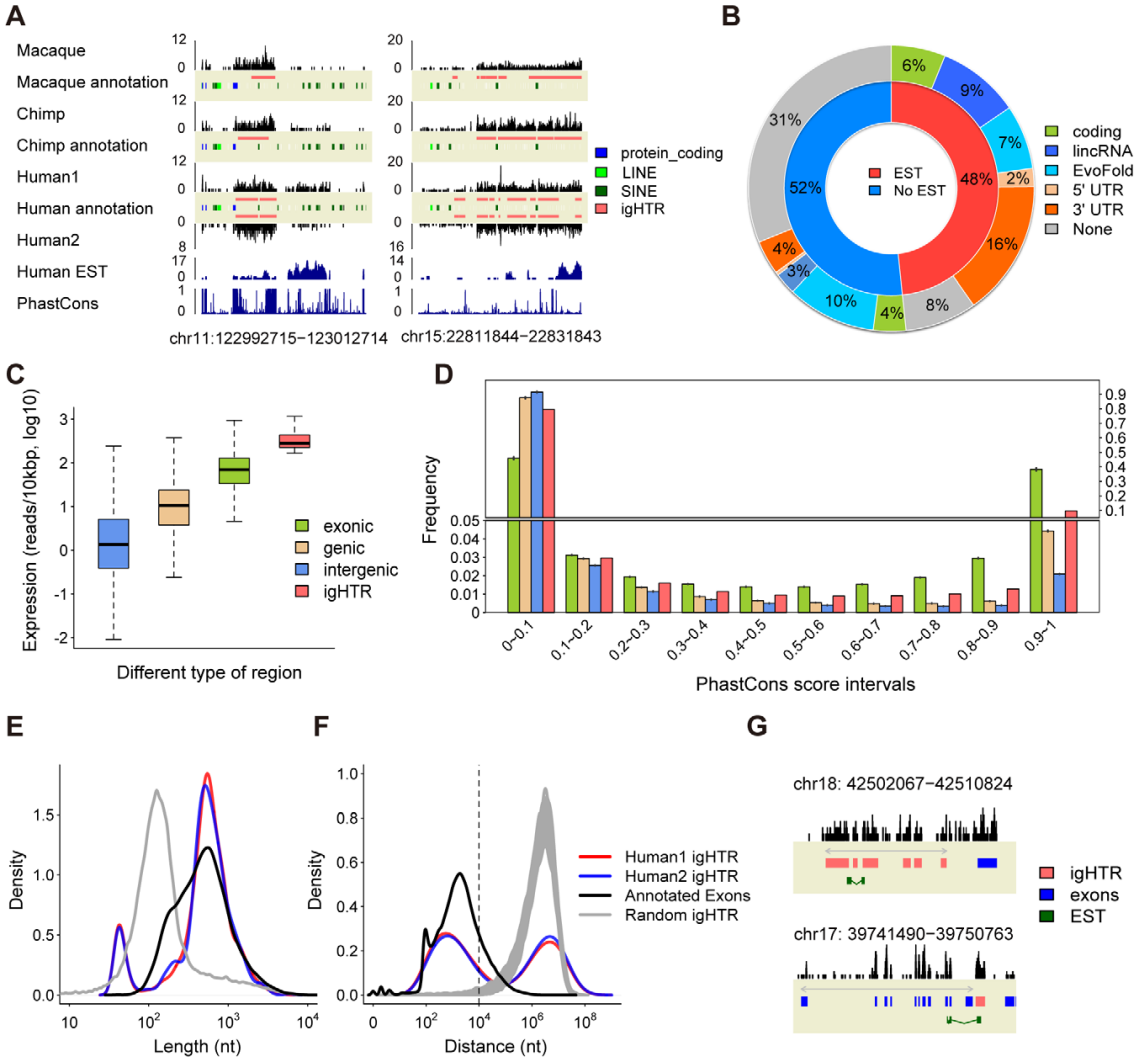
Characteristics of intergenic transcripts. (A) Examples of igHTR. The black track shows sequence reads density (in counts) in the four samples studied. The blue tracks show human EST density and PhastCon scores. (B) igHTR categories. The inner circle shows the proportion of igHTR with (red) or without (blue) EST support. The outer circle shows proportions of igHTR with protein-coding potential (green), supported by lincRNA (blue) or EvoFold (light blue) ncRNA predictions, adjacent to gene's 5′-UTR (light orange) or 3′-UTR (orange), and uncharacterized igHTR (grey) among EST-supported and non-supported igHTR. (C) Expression levels within intergenic regions (blue), genic regions including both exons and introns (light orange), exons (green), and igHTR (red). (D) Sequence conservation of nucleotides in human exons, genic regions, intergenic regions, and igHTR (all colors as on the panel (C)) based on phastCon scores among 18 placental vertebrates genomes. PhastCon scores close to 1 indicate high conservation. The heights of the bars show mean value and error bars show 95% confident intervals based on sampling of the same number of nucleotides as located within igHTR from the corresponding genomic regions 1,000 times. For igHTR, the values are based on all nucleotides located within them. (E) Size distributions of igHTR in the two human samples (red - Human1, blue - Human2), annotated human exons (grey), and exonic HTR (black) (F) Distributions of genomic distances between nearest pairs of igHTR (red – Human1, blue – Human2), annotated exons (black), and simulated randomly distributed igHTR (grey). The dashed line shows 10 kb distance. (G) Examples of splicing within igHTR clusters (red) and between annotated genes (blue) and downstream igHTR supported by EST (green).

Excluding repeats, intergenic regions contain 7% of all non-ribosomal human brain transcriptome sequences. These sequences are not distributed evenly, but concentrate within distinct regions (Figure 2A, 2B). Notably, the expression levels of such intergenic highly transcribed regions (igHTR) are comparable and, frequently exceed the expression levels of annotated exons (Figure 2C). We used two parameters to define igHTR: the maximum spacing between two neighboring reads and the minimum number of mapped sequence reads within the genomic regions. For convenience, we set these parameters to 150 nt and 10 reads for most of the analysis. In the two human samples, we find 883 and 790 of such highly transcribed intergenic regions (igHTR) not overlapping with any annotated human transcripts (Materials and Methods, Table S3). Out of these igHTR, 580 (66% and 73% for the two human samples) overlap between the samples, with the majority of igHTR overlapping by more than 80% of their length (Figure S4), while less than 1% would be expected to overlap by chance (simulation, *p*<0.01). Further, for all 1,093 igHTR identified in at least one of the two human samples, the expression levels correlated well between the samples (Spearman correlation, *rho* = 0.90, *p*<10^−15^) (Figure S5), even when the corresponding region did not pass the igHTR definition cutoff in one of the samples. Using different igHTR definition cutoffs, we get principally the same results throughout the analysis (*e.g.* Figure S6). Finally, using human brain expressed sequence tag (EST) libraries, we find further support for 48% of 1,093 igHTR found in at least one of the two human samples, significantly more than expected by chance (simulation, *p*<0.01) (Figure 2B).

Similar to humans, we can identify igHTR in chimpanzee and rhesus macaque brain transcriptomes. Expression levels of individual igHTR show significant positive correlation between the two human samples and among the three species (Spearman correlation, *rho*>0.7, *p*<10^−15^) (Figure S5). Thus, igHTR expression is largely conserved across the three primate species. To test whether igHTR are conserved at the DNA sequence level, we used PhastCons scores based on nucleotide conservation among 18 placental vertebrates genomes [14]. We find that igHTR show significantly greater conservation than randomly chosen intergenic regions or annotated genic regions including both exons and introns, but are less conserved than exons alone (Figure 2D). Further, DNA sequence conservation correlates positively with igHTR expression level (Figure S6). Thus, although both expression level and DNA sequence conservation do not prove functionality, it is likely that at least some of the identified igHTR represent functional transcripts.

Do igHTR represent extensions of known genes or independent coding and/or non-coding transcripts? The size distribution of transcription clusters shows two distinct peaks: a minor one at 45 nt and a major one at 500 nt (Figure 2E). Although 500 nt is longer than the average exon size in humans, the definition of igHTR boundaries by our method is not precise. When we define exons using the same criteria as igHTR, we find a similar length distribution for both exons and long igHTR (Figure 2E). More than half of all igHTR (65%) cluster within intergenic regions, with an average of four igHTR per group. Notably, the distances between igHTR within such clusters are similar to an average intron length (Figure 2F). Furthermore, within clusters, individual igHTR are expressed at similar levels, resembling expression of exons within a gene (Figure S7). Finally, 53 individual igHTR within clusters can be connected by at least one EST sequence, while less than 5, on average, are expected by chance (simulation, *p*<0.01) (Figure 2G, S8). Thus, more than one half of igHTR appear to form long transcripts with exon-intron structure closely resembling annotated protein-coding genes.

With respect to the genomic location, igHTR tend to be situated within gene-rich regions, with 49% of human igHTR located within 10 kb of the nearest gene (simulation, *p*<0.01). Interestingly, 84% of these igHTR are close to the 3′-end, rather than 5′-end of the nearest gene (Figure 2B). Expression levels of these igHTR correlate positively with expression of the adjacent genes (Figure S9). Further, a total of 70 out of the 452 igHTR and igHTR clusters located within 10 kb from 3′-end of the nearest gene in at least one human sample can be connected to the gene by 263 EST sequences (simulation *p*<0.01) (Figures 2G, S10). Notably, within these igHTR, we find a significant excess of conserved microRNA (miRNA) binding sites, one of the characteristic features of 3′-UTRs of annotated transcripts (Figure S11). Thus, these igHTR may represent alternative or extended 3′-UTR of annotated genes, potentially contributing to microRNA-directed expression regulation in the primate brain.

With respect to function, 251 genes that contain igHTR within 10 kb from the gene boundaries (204 of them are situated downstream for gene and may correspond to 3′-UTR extensions) show significant enrichment among GO terms [15] and KEGG pathways [16] (Table S4, S5). Notably, these genes are mainly involved in neural functions, such as signal transduction, regulation of synaptic plasticity, learning, glutamate signaling pathway and long-term potentiation pathway, as well as two major pathways associated with lifespan duration: insulin signaling and mTOR signaling.

With respect to protein coding capacity, as determined by codon substitution frequencies (CSF) [17], igHTR scored lower than known protein coding genes, but still significantly higher than known non-coding RNAs (ncRNAs) (Wilcoxon test, *p*<2.2e-16) (Figure S12). Based on the chosen CSF cutoff, approximately 10% of all human igHTR may have protein-coding capacity. The remaining igHTR may represent as yet unannotated ncRNA. Supporting this suggestion, we find significant overlap (Figure S13, simulation, *p*<0.01) between igHTR and large intergenic non-coding RNA (lincRNA) identified in mouse and human cell lines [18], [19], involving 19% of all identified human igHTR. An additional 10% of human igHTR overlap with ncRNA predictions based on secondary structure and folding potential score determined by EvoFold [20] (Figure S14, simulation, *p*<0.01) (Figure 2B).

### Transcription divergence

**Figure 3:**
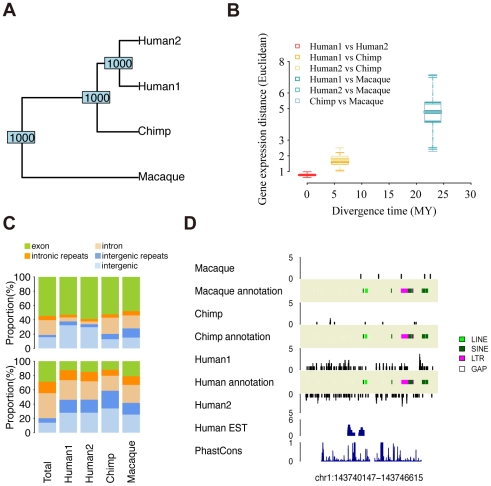
Transcription divergences. (A) UPGMA tree based on the expression level of 13,832 genes in 4 sample pools. The numbers at the nodes indicate node stability in 1,000 bootstraps over genes. (B) The gene expression divergence between sample pairs plotted against the species divergence time. The box plot represents variation of the divergence estimated from 1,000 bootstraps over genes (same set of genes as (A), see Materials and Methods). (C) The upper panels show genomic annotation of nucleotides covered by at least one sequence read within all HTR identified in at least one sample (Total) and HTR with species-specific expression. Genomic locations of species-specific HTR are listed in Table S8. The lower panels show genomic annotation of nucleotides covered by at least one sequence read within all genomic windows (Total) and genomic windows with species-specific expression. Locations of species-specific genomic windows are listed in Table S10. The colors represent: exons (green), intronic repeats (orange), introns (light orange), intergenic repeats (blue), and intergenic regions (light blue). (D) An example of a genomic window with human-specific expression.

To determine the extent of expression divergence between human, chimpanzee, and rhesus macaque brain transcriptomes, we first tested whether expression of known protein coding genes could separate species according to their phylogenetic relationship. Based on expression of 13,832 genes detected in at least two out of four samples in our dataset, we found that in agreement with previously reported results based on microarray data, gene expression differs significantly among the three species (Figure 3A, S15). Furthermore, expression divergence among species increases with the time of species divergence, independent of normalization procedures and distance measures used (Materials and Methods, Figures 3B, S16).

Next, we identified genes with species-specific expression using a Bioconductor package for differential expression analysis of digital gene expression data, “edgeR” [21]. Following this methodology, we first used the variation between two human samples to build a null model of changes in read counts across all loci studied and then used this null model to identify expression differences between species. Further, we used Benjamini-Hochberg multiple testing correction to set the false discovery rate below 5% (Materials and Methods). Following this procedure, we identified 118 genes with human-specific expression in both human samples (Table S6). To test whether these expression differences are reproducible, we compared them with published expression differences measured between three human and three chimpanzee cerebellar samples using microarrays [22]. For 34 genes present in both datasets (Materials and Methods), we find significant positive correlation of human-chimpanzee expression differences (Pearson correlation *r* = 0.68, *p* = 0.0001; Spearman correlation *rho* = 0.55, *p* = 0.0008).

Functional analysis of the 118 genes with human-specific expression did not yield significant results, but showed an enrichment trend among genes involved in transcriptional regulation (Table S4). This finding is consistent with previous studies, suggesting transcriptional regulation may play an important role in human brain evolution [23], [24], [25]. Further, in terms of amino acid divergence between humans and chimpanzees or between humans and mice, as well as promoter sequence divergence, 118 genes showed tendency for greater conservation than all genes expressed in at least one of our four samples (Table S7). Thus, observed gene expression changes are not likely to reflect relaxation of selective constraint.

In addition to gene expression differences, we compared the extent of expression divergence among the three species for different types of transcripts: exonic, intronic, intergenic, and repeats. To compare expression divergence of these different transcript types on the same basis, we used two approaches. In the first approach, in addition to igHTR, we identified all other highly transcribed regions (HTR) present in human, chimpanzee, and rhesus macaque brain transcriptomes and compared their expression levels across species. From a total of 16,159 HTR found among the three species, 10,654 (65.9%) correspond to exons, 904 (5.6%) to introns, 528 (3.3%) to intergenic regions, 3,007 (18.6%) and 1,066 (6.6%) to intronic and intergenic repeats, respectively. To identify the HTR with species-specific expression, we applied the methodology described above, based on the edgeR package. Following this approach, 24 HTR (11% in all species-specific HTR) can be classified as human-specific, 32 (15%) as chimpanzee-specific, and 159 (74%) as specific to rhesus macaque in the three species comparison (Table S8). Intriguingly, for humans, we find a slight but significant excess of HTR with species-specific expression within intergenic regions (one-sided binomial test, *p*<2.2e-16) (Figures 3C, S17, Table S9).

In the second approach, we identified regions showing extreme species-specific divergence by comparing transcriptome coverage in a sliding window over the entire human-chimpanzee-macaque (HCM) genome alignment (Figure 3D). Windows were defined to contain the same total number of sequence reads (*N* = 50) summing over all three species. Using the described above approach to identify species-specific genomic windows (GW) (Table S10), we find a strong excess of intergenic region representation in all three species (one-sided binomial test, *p*<2.2e-16) (Figure 3C lower bars, Figure S18, Table S11). We obtain the same result using both species-specific and human-based annotations (Figure S18, Table S11). Further, the result did not depend on recent duplication events or alignment problems, as determined by allowing multiple-location mapping, use of alternative reference species in alignment construction and visual inspection of all species-specific widows. Thus, in the three primate species studied, genomic regions with extreme species-specific expression patterns are more than twice as likely to originate within intergenic regions than expected by chance (Table S11).

## Discussion

Our study, although based on a few samples, uncovers basic features of the brain transcriptome that are shared among the three primate species and identifies the most divergent expression patterns specific to the human brain. Among shared features, we find that exons alone contribute approximately a quarter of the total non-ribosomal transcriptome, while exons and introns together contribute three-quarters. Previously published human brain transcriptome sequencing data based on polyadenylated transcripts contains a higher proportion of exonic and a lower proportion of intronic transcription (54% and 24%, respectively, Figure 1A) [26]. Thus, many of the intronic transcripts detected in our study may represent unprocessed non-polyadenylated precursors of mature mRNA. Non-repetitive intergenic transcripts, however, occupy similar proportions (7%) in both poly(A)-enriched and the total human brain transcriptomes.

While 42% of the human brain transcriptome originate within repetitive elements, most of the repeat expression is directly proportional to the occupied genomic length and, therefore, might represent “transcriptional background”. Some of the repeat families, however, are transcribed above the background level. While some of these families, such as snRNAs, snpRNAs and 7SK RNA that derived from functional ncRNA might be actively transcribed, high expression of simple and low complexity repeats is unusual. Notably, analysis of cap-selected mouse and human transcript tags across 12 tissues shows that simple and low complexity repeats have distinct tissue-specific expression profiles and are highly expressed in brain in both species [13]. Similarly, elevated expression of Alu elements from the most recently expanded subfamilies is unusual and indicates that these elements might be transcribed actively.

Besides repeats, intergenic transcription is highly non-uniform, containing distinct highly transcribed regions conserved between species both in terms of their expression and DNA sequence. A substantial proportion of these regions (23%) may represent alternative or extended 3′-UTR of known genes, enriched in conserved microRNA binding sites. In mouse brain, 3′-UTR extensions containing miRNA binding sites were found in microRNA-Argonaute complexes, indicating their role in miRNA-directed expression regulation [27]. Further, changes in 3′-UTR length have been shown to play a role in miRNA regulation of cell proliferation and mouse embryonic development [28], [29]. Thus, identified novel 3′-UTR may play an important role in microRNA-directed regulation in the primate brain.

Another substantial proportion of identified intergenic transcripts (29%) overlap recently identified lincRNA and ncRNA predicted by EvoFold. Since our analysis is limited to highly expressed transcripts, most of them are expressed at higher levels than protein-coding genes. This indicates that at least some of these intergenic transcripts represent novel ncRNA functioning in the primate brain. We have to note, however, that these transcripts represent a small fraction of all identified lincRNA and ncRNA predicted by EvoFold: 1.7% and 0.3%, respectively. Thus, the vast majority of lincRNA and ncRNA predicted by EvoFold are not expressed in human cerebellum, or are expressed at levels below our igHTR detection threshold.

With respect to evolutionary features, the extent of expression divergence increases with greater species' phylogenetic divergence time. In our study, we do not observe an excess of expression divergence on the human lineage, previously reported in another brain region, cerebral cortex [6], [7]. Thus, in different brain regions, the transcriptome may have evolved at different rates during human evolution. It has to be noted, however, that our study does not provide intra-species variation estimates, and cannot be directly compared with the previous studies. Further work is needed to investigate this question. Notably, we find that the most extreme human-specific expression patterns, as well as extreme expression patterns characteristic for the other two primate species, show greater than expected enrichment within intergenic regions. Thus, further characterization of intergenic transcription will be necessary for understanding regulatory evolution in primates and identification of the molecular mechanisms underlying the evolution of the human-specific phenotype.

## Materials and Methods

### Ethics statement

Informed consent for use of the human tissues for research was obtained in writing from all donors or the next of kin. All non-human primates used in this study suffered sudden deaths for reasons other than their participation in this study and without any relation to the tissue used.

### Sample preparation and sequencing

We dissected postmortem cerebellar cortex samples from ten male humans (8–54 years old), four male chimpanzees (8–40 years old), and five male rhesus macaques (4–20 years old). All human *postmortem* brain tissue samples were obtained from the NICHD Brain and Tissue Bank for Developmental Disorders (NICHDBB) (Baltimore, MD, USA). Forensic pathologists at the NICHDBB defined all subjects as normal controls. No subjects with prolonged agonal state were used. Chimpanzee samples were obtained from the Yerkes Primate Center (Atlanta, GA, USA), the Anthropological Institute & Museum of the University of Zürich-Irchel, (Zürich, Switzerland), and from the Biomedical Primate Research Centre (Rijswijk, Netherlands). The rhesus macaque samples were obtained from the SuZhou Experimental Animal Center (SuZhou, China). All samples contained RNA of comparable and high quality (Table S1).

Total RNA was extracted from dissections by Trizol reagent (Invitrogen, Carlsbad, CA) and treated for 30 min at 37°C with RNase free DNase I (Ambion, Austin, TX). RNA was purified with the RNeasy MinElute Kit according to the manufacturer's instructions (Qiagen, Valencia, CA). This procedure depletes RNA molecules with length shorter than 200 nt. Resulting RNA samples from five human, five macaque, or four chimpanzee individuals was mixed in equal proportions within species, resulting in two human, one chimpanzee, and one rhesus macaque pooled samples (Table S1). 10ug RNA was treated with two rounds of RiboMinus kit (Invitrogen) to remove most of the Ribosome RNA. The cDNA libraries were prepared starting from 2ug of rRNA-Reduced total RNA per sample and using random hexamer primers (Invitrogen, Cat. No. 48190-011). It has to be noted that the resulting double-stranded cDNA fragments do not preserve information about the strand specificity of the original transcript. The Illumina sequencing libraries were prepared according to the single-end sample preparation protocol (http://www.illumina.com). The libraries were sequenced using the 1G Illumina Genome Analyzer. The sequencing products were the single-end 36 nucleotides (nt) long sequence reads. All sequence data including quality scores is deposited into the NCBI's Short Read Archive, accession number SRA011534.

### Read mapping and annotation

All raw sequencing reads were mapped to the corresponding reference genomes (hg18, panTro2, and rheMac2), allowing a maximum of four mismatches, using Short Oligonucleotide Alignment Program (SOAP, version 1) [30]. Using a smaller number (two or three) of allowed maximum mismatches did not affect the analysis (data not shown). Only the reads that mapped uniquely were included in the analysis, unless indicated otherwise. For the three species, all uniquely mapped reads were annotated based on the species-specific gene annotation from Ensembl (release 50) provided by BioMart (http://www.biomart.org/) [31] or based on the human annotation (see below). Throughout the analysis, exons and intron categories are based on all exons, both coding and non-coding, of protein-coding genes according to Ensembl (release 50) annotation. Reads mapping to rRNA (both uniquely and allowing multiple mapping) were excluded from the analysis. To ensure complete and unbiased exclusion of rRNA sequences, for each species we mapped reads to all rRNA sequences annotated in the three species. In each sample, 36–39% of all mapped reads mapped to rRNA. For uniquely mapped reads, 1–2% mapped to rRNA. The repeat annotation was taken from the RepeatMasker table provided by the UCSC table browser (http://genome.ucsc.edu/) [32]. The genomes were separated into 7 categories: exons, intronic repeats, introns, intergenic repeats, mitochondrial chromosome, non-coding RNA, and intergenic regions. This order is further used as a category hierarchy for sequence reads annotation, from the highest to the lowest level, respectively. A sequence read was assigned to a category if at least one nucleotide of the read mapped to the category's genomic region according to the above hierarchy and independent of the strand orientation, as strand information was lost during sequence library preparation. Further, sequence reads mapped to exon junctions were assigned to exons. Although our approach biases annotation to the categories high in the hierarchy, such as repetitive elements, this effect is not large. Specifically, we find that in humans only 7% of all sequence reads we assign to repeats do not map completely within repetitive elements and, therefore, can be assigned to other categories. Further, for only 2% of all sequence reads we assign to repeats, less than half of a read sequence is contained within repetitive elements. The distribution of mapped reads shown in (Figure 1A) and (Figure S1A) is based on counting the number of sequence reads mapped to both unique and multiple (≤100 locations) positions in the genome. We obtain similar results considering only sequence reads mapped to unique genomic positions (Figure S1B).

### Repeat transcription

We estimated the expression levels of repeat families based on uniquely mapped sequences. Including sequence reads mapped to multiple positions increased the total number of reads mapped to repeat regions by approximately 10%, but did not affect the results qualitatively. To normalize the expression by the lengths of unique DNA in each repeat family, we calculated the numbers of potential positions in repeat elements that can be mapped uniquely, then we summed up these numbers of all the elements and that of the expressed elements separately. This length calculation was done for both the analysis of repeat expression level *vs.* repeat length (Figure 1B) and the analysis of repeat transcriptional activity *vs.* repeat age (Figure 1C, 1D).

### Three-species genome alignment

Pair-wise genome alignments of human-chimpanzee and human-macaque were downloaded from UCSC genome browser (genome versions: hg18, panTro2 and rheMac2). Based on these alignments, Human-Chimpanzee-Macaque (HCM) three-way alignment was constructed Using Multiz software package [33]. The human genome was selected as reference during the construction unless indicated otherwise. The regions in the HCM alignment were also considered as 3 species consensus regions (HCM consensus regions).

### HTR definition and analysis

We used two parameters to determine whether a region is a HTR. The first is the maximum spacing (maxspacing) between two neighboring reads (from 5 to 3′ on the forward strand). The second is the minimum number of mapped sequences (minhits) within the regions. For convenience, we use maxspacing = 150 nt and minhits = 10 for all HTR analysis shown in the paper, except Figure S5. The chosen parameters are conservative, as we only select genomic regions with unusually high expression levels (Figure 2B). Using other parameter sets did not affect the results. igHTR were defined to be located entirely within intergenic regions according to Ensembl (release 50) annotation of protein-coding genes, non-coding genes, and pseudogenes. Further, they did not overlap with RefSeq and VEGA transcript annotation (downloaded from UCSC table browser, http://genome.ucsc.edu) [32]. To identify HTR in humans, chimpanzee, and macaque on an equal basis, total numbers of mapped sequences were equalized among the samples by random sub-sampling of 1,500,000 mapped sequences for each sample. This number was based on the read number in the sample with the lowest coverage. HTR were aligned across species based on HCM alignment. HTR genomic boundaries were defined based on the 5′-most and 3′-most coordinates found among the four samples.

To calculate the expression correlation of individual igHTR in the three species, we unified igHTR identified in the four samples. We mapped igHTR identified in chimpanzee and macaque onto the human genome using the LiftOver tool from UCSC genome browser (http://genome.ucsc.edu/cgi-bin/hgLiftOver).

All simulation tests were done by randomly selecting the same number of genomic regions with the same length distribution as the actual igHTR 1,000 times. The sample genomic regions differed depending on the tested variable and are described specifically in each case (see Supplementary Information for details).

Sequence conservation analysis was based on the sequence conservation measures provided for each nucleotide position by the PhastCons conservation scores for 18-way multiple alignments between the human genome and 17 other placental mammalian species [34]. Conservation was determined for nucleotides within human igHTR, as well as for the entire human intergenic regions, genic regions (including both exons intron), and exons by randomly sampling the same number of nucleotides from these regions 1,000 times.

We tested protein-coding potential of human igHTR by determining the maximum CSF (codon substitution frequency) score observed across the entire genomic locus, following [17]. Briefly, we used a scoring matrix built from human-mouse alignment and computed the CSF scores across sliding windows of 90 nucleotides. We then scanned all 6 possible reading frames in each window, since we have the strand information. After computing a score for each window, we defined the “max CSF score” for a cluster to the maximum observed score across the region. Then, we chose CSF cutoff that discriminates well between coding and non-coding regions based on the CSF distributions of known protein-coding and non-coding regions. We chose cutoff at CSF = 2, which gives specificity (97.9%) and sensitivity (93.2%) (Figure S12). Finally, we applied this cutoff to the CSF distributions of igHTR to estimate the proportion of potential protein-coding regions.

For overlap between lincRNA (large intergenic non-coding RNA) and igHTR, we used published lincRNA identified in mouse [18] and human [19]. We downloaded the lincRNA tables provided by these two papers and identified the human orthologs of the mouse lincRNA as described in [19]. Next, we combined the human lincRNA and the human orthologs with mouse lincRNA for the analysis.

For overlap between EvoFold predictions and igHTR, we download a total of 47,510 predicted RNA from UCSC browser [20]. As many of these predictions are short (∼20 nt), we assume that they originate from a longer precursor and extend the predicted locations by 1 kb at both ends for the analysis.

### Transcription divergence

Among all annotated human protein-coding genes (Ensembl release 50), 18,391 can be matched between the three species based on HCM alignment. Out of these genes, 13,832 expressed in at least two of the four samples were used in this analysis. The gene expression levels were calculated as the number of sequence reads uniquely mapped in exons, normalized by the gene's exonic region length. Reads mapped to exon junctions were not counted here, because some exon boundaries might not been matched accurately between genomes based on HCM genome alignment. The expression levels were normalized across samples using quantile normalization (normalize.quantiles function in R) [35]. Divergence between samples was estimated based on Euclidean distance, Manhattan distance, and 1-*rho* (Spearman correlation coefficient) (Figures S15, S16). Further, to remove influence of expression level on divergence calculation, we Z-transformed expression levels before the expression distance calculation: the expression level of each gene was set to mean = 0 and standard deviation = 1 across the four samples (Figures S15, S16). The UPGMA trees (Figure S15) were constructed using R-package ape and phangorn.

### Species-specific expression

To identify species-specific expression of genes, HTR, or GW, we used a Bioconductor package for differential expression analysis of digital gene expression data, “edgeR” [21]. This package models the digital expression data using a negative binomial (NB) distribution with parameters estimated from the actual data. First, we estimated the dispersion parameter in the NB model by comparing expression in two human samples (function estimateCommonDisp in edgeR package). This estimated common dispersion was then used in an exact test (function exactTest) analogous to the Fisher's exact test to detect differential expression between any two species. The resulting *p*-values were adjusted with Benjamini-Hochberg multiple testing correction to control the false discovery rate to be below 5%. Species-specific expression was identified separately in two groups of samples. Group one (G1) contained Human1, Chimpanzee, and Macaque samples. Group two (G2) contained Human2, Chimpanzee, and Macaque samples. Genes, HTR, or GW with significant expression difference in human-chimpanzee and human-macaque comparisons, but not in chimpanzee-macaque comparison, in both G1 and G2 were classified to have human-specific expression. Similarly, we identified genes, HTR, or GW with chimpanzee-specific and rhesus macaque-specific expression (Tables S6, S8, and S10).

HTR were determined over the entire HCM alignment using standard parameters (maxspacing = 150 nt and minhits = 10) and assigned to the annotation categories according to the hierarchy mentioned above (Materials and Methods: Read mapping and annotation). We defined GW as HCM alignment regions containing a total of 50 sequence reads in the three species.

### GO/KEGG enrichment analysis

For 118 genes with human-specific expression, 251 genes containing igHTR (within 10 kb from the gene boundaries in both directions in the human samples), and for 204 (of 251) genes with igHTR near 3′-UTR, we performed GO-term/KEGG-pathway enrichment analysis using 15,263 genes expressed in at least one out of four samples as background. For the GO function enrichment analysis, we downloaded the Ensembl gene-GO annotation from the Ensembl database [31]. We then used the func_hyper program of the package FUNC to test for category enrichment. The program generates raw enrichment *p*-values for each category based on hypergeometric distribution, then performs permutations of genes to determine whether the detected enrichment is greater than expected by chance, generating a global enrichment *p*-value [36]. For KEGG pathway enrichment analysis, we downloaded Ensembl gene-KEGG annotation from the KEGG database, and use in-house code written in R-language (supplied on request) that uses the same strategy as func_hyper. The resulting GO terms from “biological process” taxonomy and KEGG pathways with raw enrichment *p*-value<0.05 are listed in Tables S4 and S5.

### Comparisons with published data

To compare human-chimpanzee expression differences, we used expression data measured using Affymetrix arrays in three human and three chimpanzee adult cerebellar samples [22]. Provided expression levels of 6,645 genes were quantile normalized and log2 transformed. Based on these data, for each gene we calculated human-chimpanzee difference as the difference between mean expression levels in the two species. In our current RNA-Seq data, 14,959 genes are expressed in at least one of the three samples. For these genes, we quantile normalized the expression levels across three samples, log2-transformed, and calculated human-chimpanzee difference as the difference between mean expression levels in the two species. Out of 118 genes with human-specific expression in RNA-Seq experiment, 34 were present in both data sets.

### Conservation of the human-specific genes

We compare selective constrains in 118 genes with human-specific expression to that of 15,263 genes expressed in at least one out of four samples based on three measures: (1) Ka/Ks between human and mouse: the data was downloaded from Ensembl (release 50) [31] via Biomart and only considering 1∶1 orthologs between human and mouse. (2) Ka/Ki between human and chimpanzee: this data was downloaded from [37]. (3) Promoter sequence divergence (Kp) between human and chimpanzee: this data was downloaded from [38]. The results are shown in Table S7.

## Supporting Information

Figure S1 Composition of primate brain transcriptome
(0.14 MB DOC)

Figure S2 DNA length and transcriptional activity of repeat families
(0.25 MB DOC)

Figure S3 Relationship between age rank and transcriptional activity of transposable element families
(0.15 MB DOC)

Figure S4 The proportion of igHTR overlaps between the two human samples
(0.10 MB DOC)

Figure S5 Expression correlation of igHTR
(1.29 MB DOC)

Figure S6 Distribution of DNA sequence conservation scores at different igHTR expression cutoffs
(0.67 MB DOC)

Figure S7 Correlation of expression levels of igHTR that cluster within the genome
(0.09 MB DOC)

Figure S8 Connections between igHTR within clusters supported by EST
(0.04 MB DOC)

Figure S9 Expression correlation between igHTR and the nearest protein-coding gene
(0.23 MB DOC)

Figure S10 Number of igHTR/gene 3 prime end connections verified by at least one EST sequence
(0.15 MB DOC)

Figure S11 Number of conserved miRNA target sites in igHTR
(0.10 MB DOC)

Figure S12 Codon Substitution Frequency (CSF) score in different types of regions
(0.08 MB DOC)

Figure S13 Overlap between igHTR and lincRNAs
(0.10 MB DOC)

Figure S14 Overlap between igHTR and EvoFold predictions
(0.10 MB DOC)

Figure S15 Gene expression trees based on different measures of gene expression divergence
(0.16 MB DOC)

Figure S16 Gene expression divergence vs. evolutionary time
(0.27 MB DOC)

Figure S17 Genomic annotation of expressed regions within HTR
(0.21 MB DOC)

Figure S18 Genomic annotation of expressed regions within GW
(0.21 MB DOC)

Table S1 Sample information
(0.02 MB XLS)

Table S2 Numbers of sequence reads
(0.02 MB XLS)

Table S3 Genomic coordinates, category and EST overlap of igHTR
(0.18 MB XLS)

Table S4 GO term(biological process) enrichment analysis results
(0.02 MB XLS)

Table S5 KEGG pathway enrichment analysis results
(0.03 MB XLS)

Table S6 Species-specific Genes
(0.13 MB XLS)

Table S7 Evolutionary conservation of human specific genes
(0.02 MB XLS)

Table S8 Species-specific HTR
(0.11 MB XLS)

Table S9 Annotation of genomic DNA expressed in HTR
(0.02 MB XLS)

Table S10 Species-specific GW
(1.54 MB XLS)

Table S11 Annotation of genomic DNA expressed in GW
(0.02 MB XLS)
