# Supplementary material for: Intergenic and Repeat Transcription in Human, Chimpanzee and Macaque Brains Measured by RNA-Seq
Source: PLoS Comput Biol. 2010 Jul 1;6(7):e1000843. doi: 10.1371/journal.pcbi.1000843 (PMC2895644; doi:10.1371/journal.pcbi.1000843)
Supplement: Figure S1 — Composition of primate brain transcriptome (0.14 MB DOC) [file pcbi.1000843.s001.doc]

**Figure S1**

**A**

**
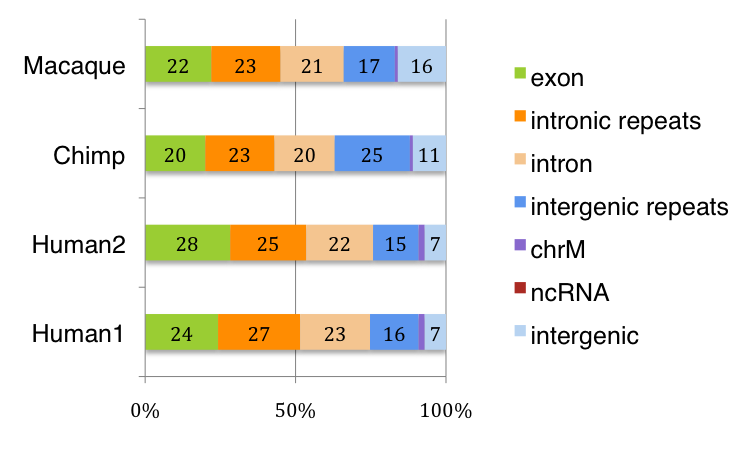
**

**B**

**
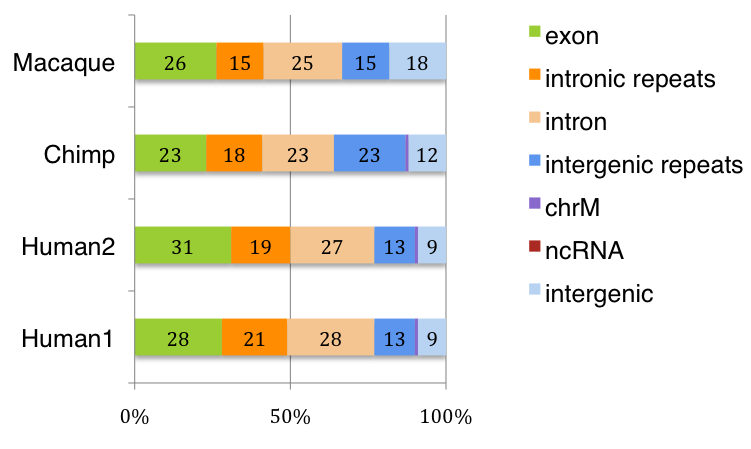
**

**Figure S1. Composition of primate brain transcriptome.** Shown in each bar are proportions of sequence reads that mapped within exons (green), intronic repeats (orange), introns (light orange), intergenic repeats (blue), mitochondrial DNA (purple), ncRNA (maroon), and intergenic regions (light blue). Sample names are indicated on the left of bars. The proportions are measured by counting the number of: (**A**) sequence reads mapped to unique and multiple (<100) locations in the genome (**B**) sequence reads mapped only to unique locations in the genome.
