# Supplementary material for: Intergenic and Repeat Transcription in Human, Chimpanzee and Macaque Brains Measured by RNA-Seq
Source: PLoS Comput Biol. 2010 Jul 1;6(7):e1000843. doi: 10.1371/journal.pcbi.1000843 (PMC2895644; doi:10.1371/journal.pcbi.1000843)
Supplement: Figure S2 — DNA length and transcriptional activity of repeat families (0.25 MB DOC) [file pcbi.1000843.s002.doc]

**Figure S2**

**A**


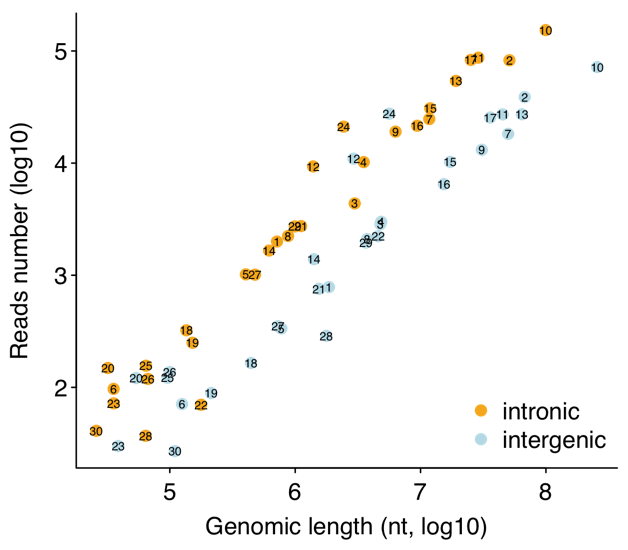


**B**

**
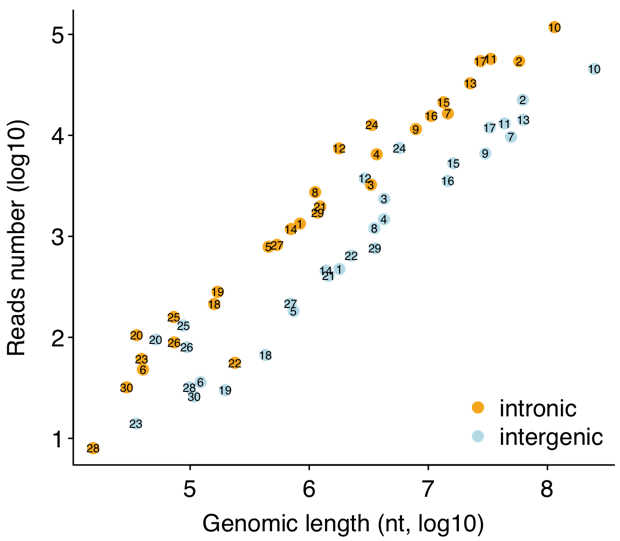
**

**C**

**
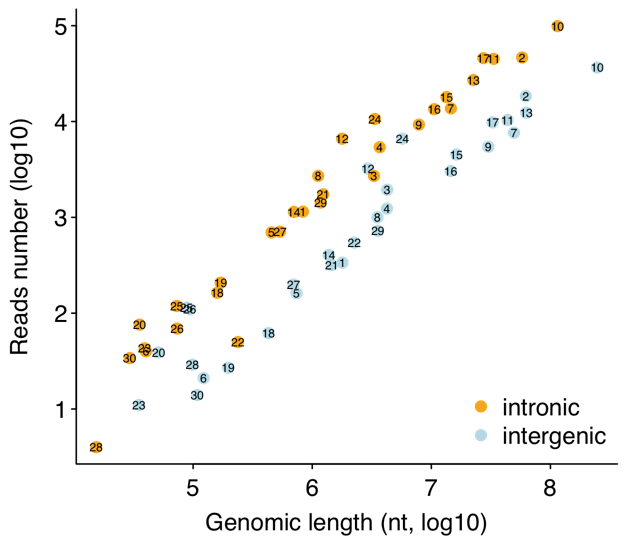
**

**D**

**
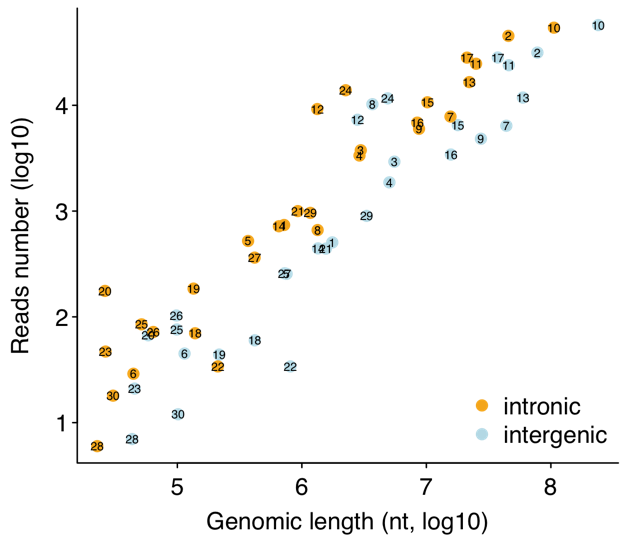
**

**Figure S2. DNA length and transcriptional activity of repeat families.** Shown on the Y-axis are the transcriptional activities of repeat families as determined by the number of uniquely mapped sequence reads plotted against the genomic length of the families, excluding genomic positions where 36 nt long sequence reads cannot be mapped uniquely. Transcriptional activity and length estimations were carried out separately for repeats located within introns (orange) and intergenic regions (light blue). Results from 4 samples were shown separately in 4 figures: (**A**) Chimpanzee, (**B**) Human1, (**C**) Human2, (**D**) Macaque. The labels represent different repeat families (1: AcHobo, 2: Alu, 3: AluY, 4: CR1, 5: DNA, 6:ERV, 7: ERV1, 8: ERVK, 9: ERVL, 10: L1, 11: L2, 12: Low_complexity, 13: MaLR, 14 Mariner, 15: MER1_type, 16: MER2_type, 17: MIR, 18: MuDR, 19: PiggyBac, 20: RNA, 21: RTE, 22: Satellite, 23: scRNA, 24: Simple_repeat, 25: snRNA, 26: srpRNA, 27: Tc2, 28: telo, 29: Tip100).
