# Supplementary material for: Intergenic and Repeat Transcription in Human, Chimpanzee and Macaque Brains Measured by RNA-Seq
Source: PLoS Comput Biol. 2010 Jul 1;6(7):e1000843. doi: 10.1371/journal.pcbi.1000843 (PMC2895644; doi:10.1371/journal.pcbi.1000843)
Supplement: Figure S3 — Relationship between age rank and transcriptional activity of transposable element families (0.15 MB DOC) [file pcbi.1000843.s003.doc]

**Figure S3**

**A B**

**
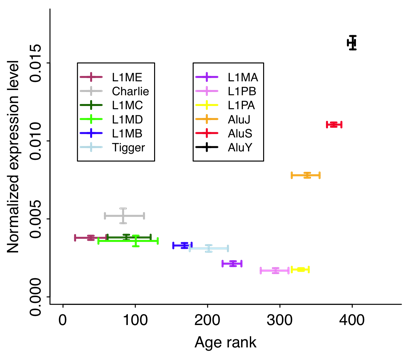

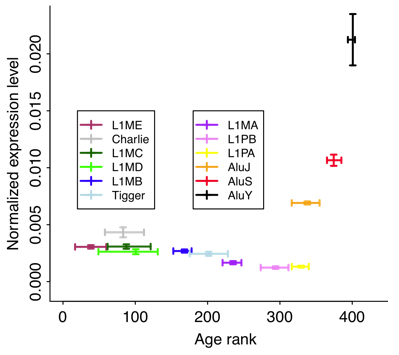
**

**C D**

**
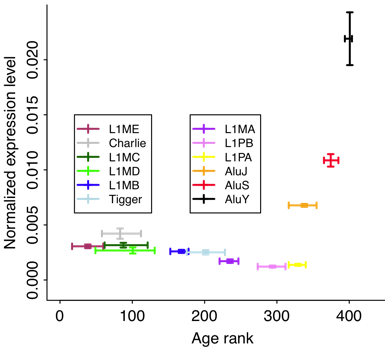

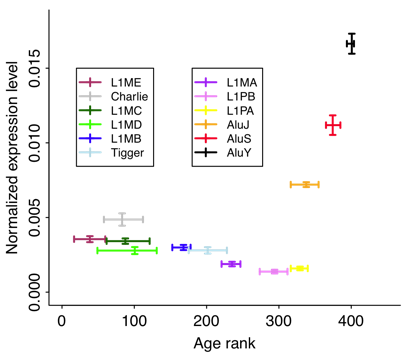
**

**Figure S3. Relationship between age rank and transcriptional activity of transposable element families.** Shown are transcriptional activities of 12 TE families plotted against their ages. Bigger age ranks correspond to evolutionary younger TE families. The horizontal bars indicate the range of estimated age rank. On the Y-axis, the transcriptional activity of transposon families is calculated as the total number of uniquely mapped sequence reads normalized by the genomic lengths of expressed repeat elements, excluding genomic positions where 36 nt long sequence reads cannot be mapped uniquely. Only repeat elements located within intergenic regions are considered for both expression level and genomic length calculations. Expression of repeat elements located within introns shows the same pattern (data not shown). The vertical bars indicate the 95% confidence intervals of expression level estimates determined by 1,000 bootstraps over expressed repeat elements within each TE family. Results from 4 samples are shown separately in 4 figures: (**A**) Chimp, (**B**) Human1, (**C**) Human2, (**D**) Macaque.
