# Supplementary material for: Intergenic and Repeat Transcription in Human, Chimpanzee and Macaque Brains Measured by RNA-Seq
Source: PLoS Comput Biol. 2010 Jul 1;6(7):e1000843. doi: 10.1371/journal.pcbi.1000843 (PMC2895644; doi:10.1371/journal.pcbi.1000843)
Supplement: Figure S4 — The proportion of igHTR overlaps between the two human samples (0.10 MB DOC) [file pcbi.1000843.s004.doc]

**Figure S4**


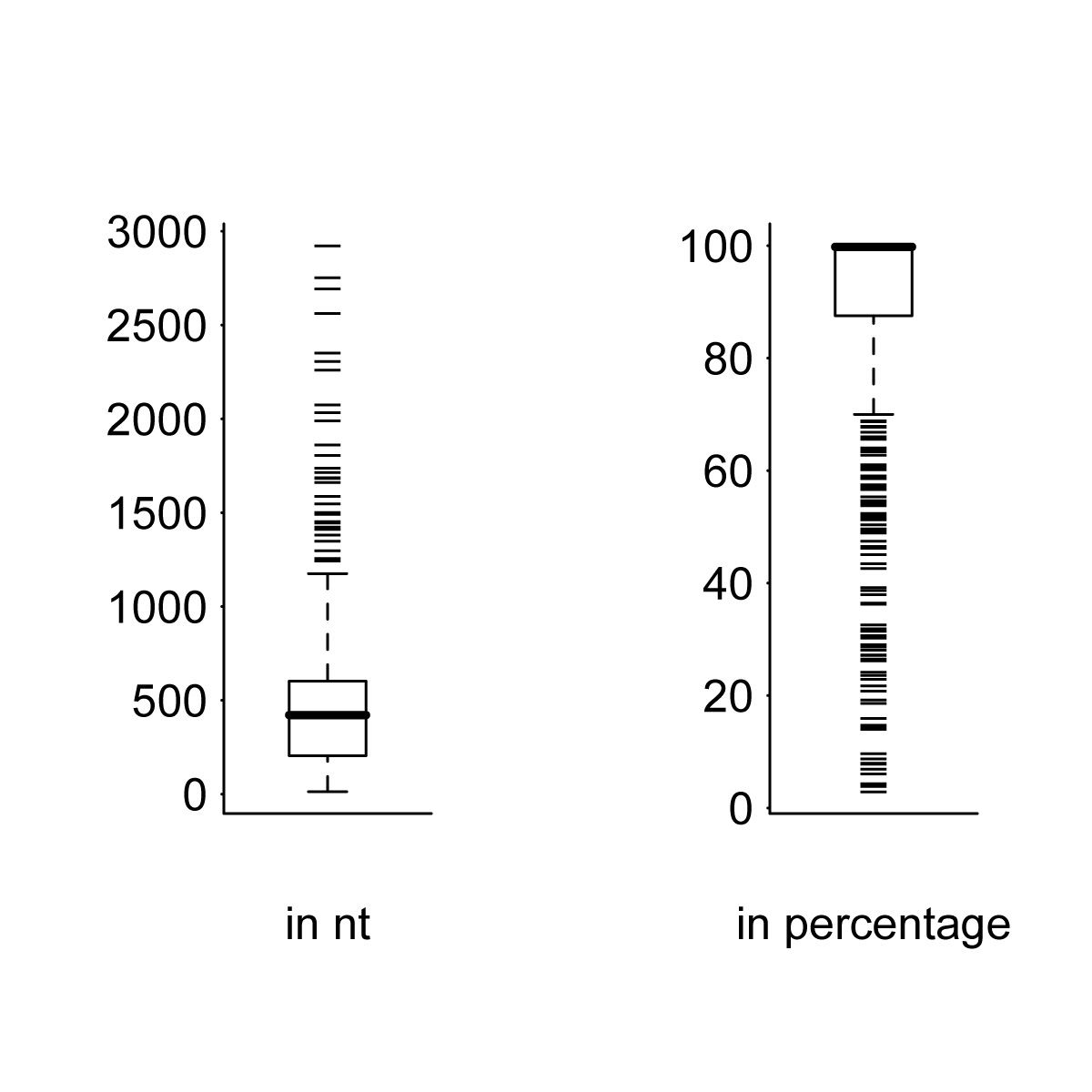


**Figure S4. The proportion of igHTR overlaps between the two human samples.** The left panel shows the overlap in number of nucleotides between igHTR identified in both human samples. The y-axis shows the overlap length in nucleotides. The right panel (y-axis) shows the overlap as a percentage of overlapping regions in the shorter one of the two overlapping igHTR.
