# Supplementary material for: Intergenic and Repeat Transcription in Human, Chimpanzee and Macaque Brains Measured by RNA-Seq
Source: PLoS Comput Biol. 2010 Jul 1;6(7):e1000843. doi: 10.1371/journal.pcbi.1000843 (PMC2895644; doi:10.1371/journal.pcbi.1000843)
Supplement: Figure S5 — Expression correlation of igHTR (1.29 MB DOC) [file pcbi.1000843.s005.doc]

**Figure S5**


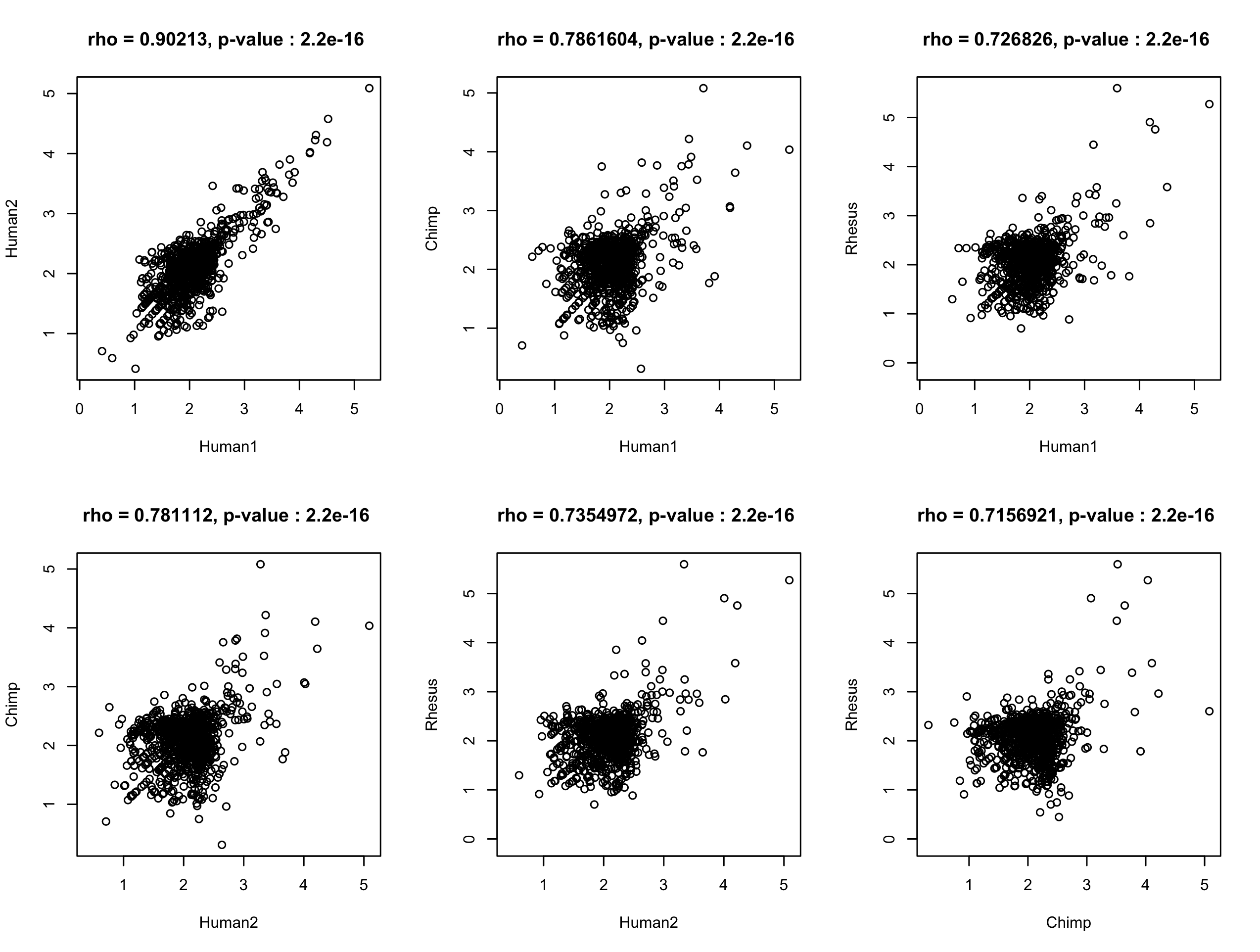


**Figure S5. Expression correlation of igHTR.** The X- and Y-axes show the expression levels of igHTR for all pairwise comparisons of the four samples. For each comparison, expression levels of igHTR identified in at east one of the two samples are shown. Sample annotations are shown on the X- and Y-axis labels. Expression levels are calculated as log10-transformed number of sequence reads mapped uniquely within an igHTR. The genomic boundaries of individual igHTR are assigned as a union of igHTR regions in all samples. Correlations are calculated using Spearman method, *rho*-and *p*-values are indicated above each plot.
