# Supplementary material for: Intergenic and Repeat Transcription in Human, Chimpanzee and Macaque Brains Measured by RNA-Seq
Source: PLoS Comput Biol. 2010 Jul 1;6(7):e1000843. doi: 10.1371/journal.pcbi.1000843 (PMC2895644; doi:10.1371/journal.pcbi.1000843)
Supplement: Figure S6 — Distribution of DNA sequence conservation scores at different igHTR expression cutoffs (0.67 MB DOC) [file pcbi.1000843.s006.doc]

**Figure S6**

**A**

**
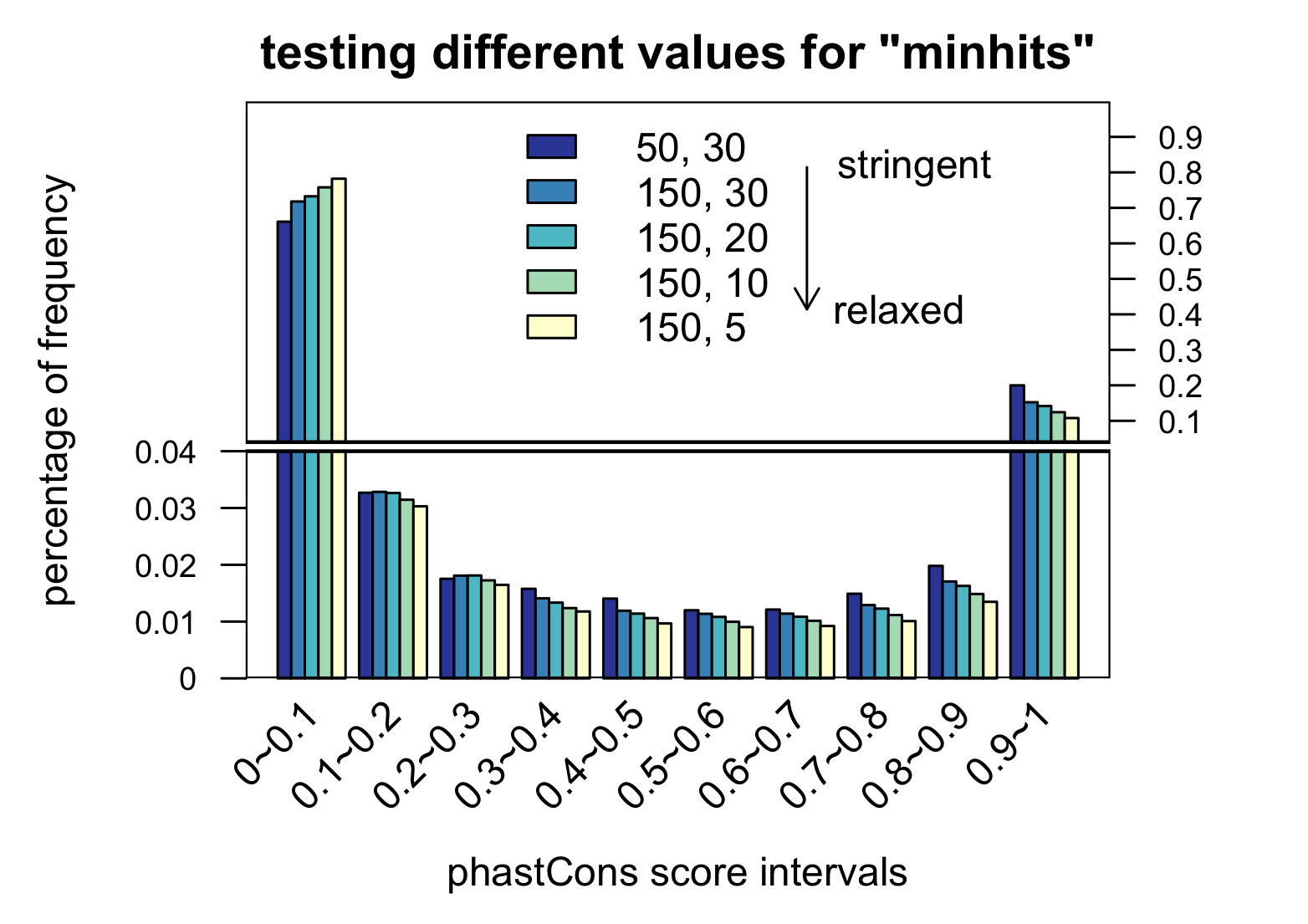
**

**B**

**
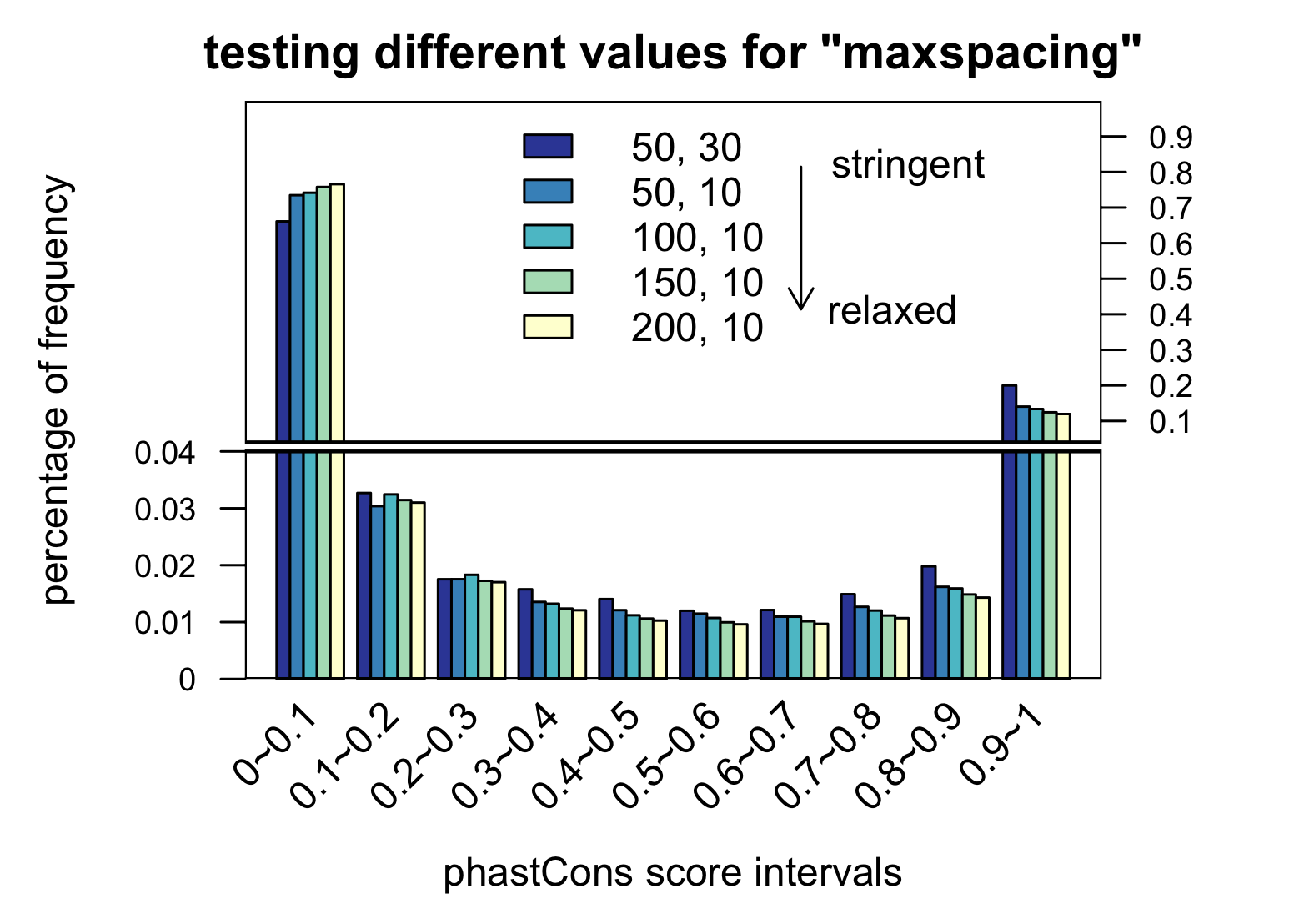
**

**Figure S6. Distribution of DNA sequence conservation scores at different igHTR expression cutoffs.** (**A**) Distribution of igHTR PhastCons scores reflecting sequence conservation (zero – no conservation, one – maximum conservation) at different igHTR sequence coverage threshold determined by varying the “minhits” parameter from stringent to relaxed (30, 20, 10, 5) while “maxspacing” stays at 150, as well as the most stringent criterion tried: the combination of “maxspacing”, “minhits” of (50, 30) (**B**) The same as (**A**), but varying the “maxspacing” parameter from stringent to relaxed (50, 100, 150, 200) while “minhits” stays at 10, and the combination of “maxspacing”, “minhits” of (50, 30).
