# Supplementary material for: Intergenic and Repeat Transcription in Human, Chimpanzee and Macaque Brains Measured by RNA-Seq
Source: PLoS Comput Biol. 2010 Jul 1;6(7):e1000843. doi: 10.1371/journal.pcbi.1000843 (PMC2895644; doi:10.1371/journal.pcbi.1000843)
Supplement: Figure S7 — Correlation of expression levels of igHTR that cluster within the genome (0.09 MB DOC) [file pcbi.1000843.s007.doc]

**Figure S7**

**
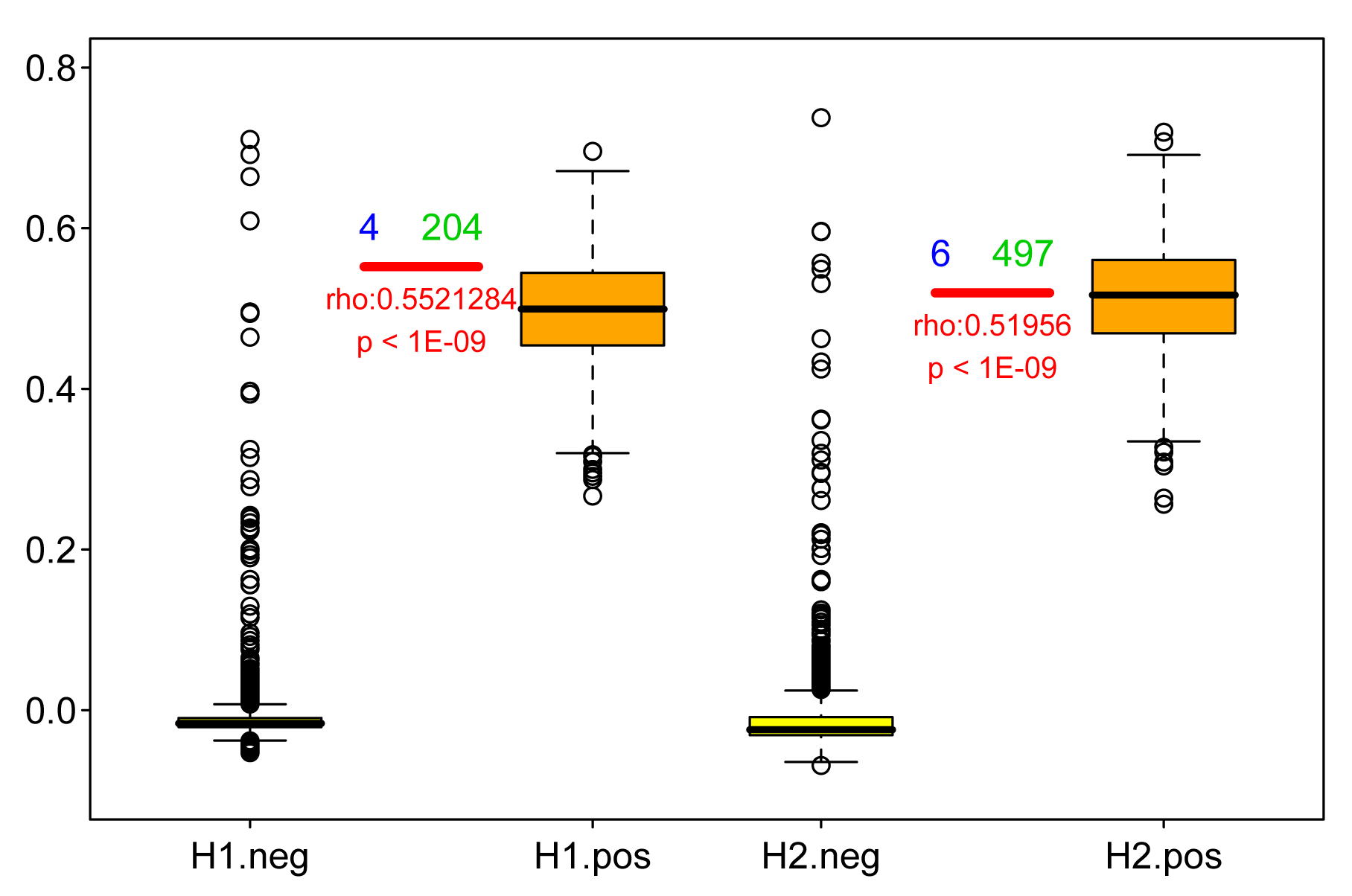
**

**Figure S7. Correlation of expression levels of igHTR that cluster within the genome.** igHTR are assigned to a distinct group when the genomic distance between two neighboring igHTR is less than 10 kb. Red lines indicate the mean Spearman correlation *rho* between expression of igHTR elements within igHTR clusters found in human sample 1 (H1) and human sample 2 (H2) (*p* < 10^-8^). Positive control (orange) shows the distribution of mean correlation coefficients found between expression levels for the same number of exonic HTR pairs as the number of clustering igHTR pairs tested, randomly sampled from expressed genes 1,000 times. Negative control (yellow) shows the distribution of mean correlation coefficients found between expression levels for the same number of randomly composed igHTR pairs that don’t fall into the same cluster as the number of clustering igHTR pairs tested, randomly sampled 1,000 times. The numbers above the red lines indicate the number of times we observed correlation greater or equal to the one found in igHTR clusters in negative (blue) and positive (green) controls out of 1,000 simulations. The boxes show variation of sampled correlation measurements and are draw using R-function “boxplot”.
