# Supplementary material for: Intergenic and Repeat Transcription in Human, Chimpanzee and Macaque Brains Measured by RNA-Seq
Source: PLoS Comput Biol. 2010 Jul 1;6(7):e1000843. doi: 10.1371/journal.pcbi.1000843 (PMC2895644; doi:10.1371/journal.pcbi.1000843)
Supplement: Figure S8 — Connections between igHTR within clusters supported by EST (0.04 MB DOC) [file pcbi.1000843.s008.doc]

**Figure S8**

**
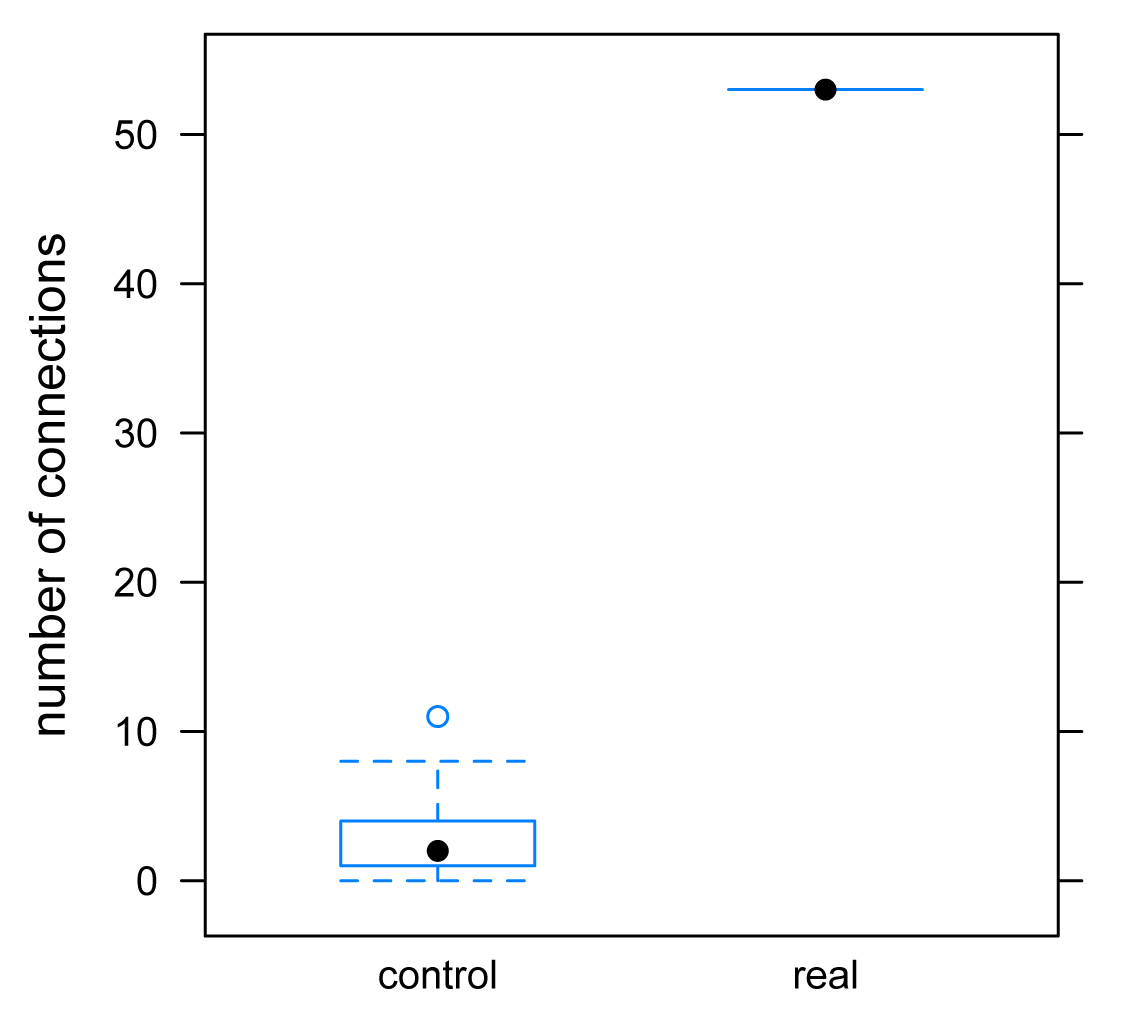
**

**Figure S8. Connections between igHTR within clusters supported by EST.** Y-axis shows number of connections between two adjacent igHTR within a cluster supported by at least one human brain EST sequence. The igHTR are called connected if EST coordinates on the human genome overlap the coordinates of two adjacent igHTR by at least one nucleotide. Control shows the number of EST-supported connections within igHTR clusters of the same length and genomic organization as the real ones randomly placed within intergenic regions 1,000 times. Real shows the number of EST-supported connections within actual igHTR clusters. The box shows variation of simulated connection measurements and are drawn using function “bwplot” in R package “lattice” with no modification.
