# Supplementary material for: Intergenic and Repeat Transcription in Human, Chimpanzee and Macaque Brains Measured by RNA-Seq
Source: PLoS Comput Biol. 2010 Jul 1;6(7):e1000843. doi: 10.1371/journal.pcbi.1000843 (PMC2895644; doi:10.1371/journal.pcbi.1000843)
Supplement: Figure S9 — Expression correlation between igHTR and the nearest protein-coding gene (0.23 MB DOC) [file pcbi.1000843.s009.doc]

**Figure S9**


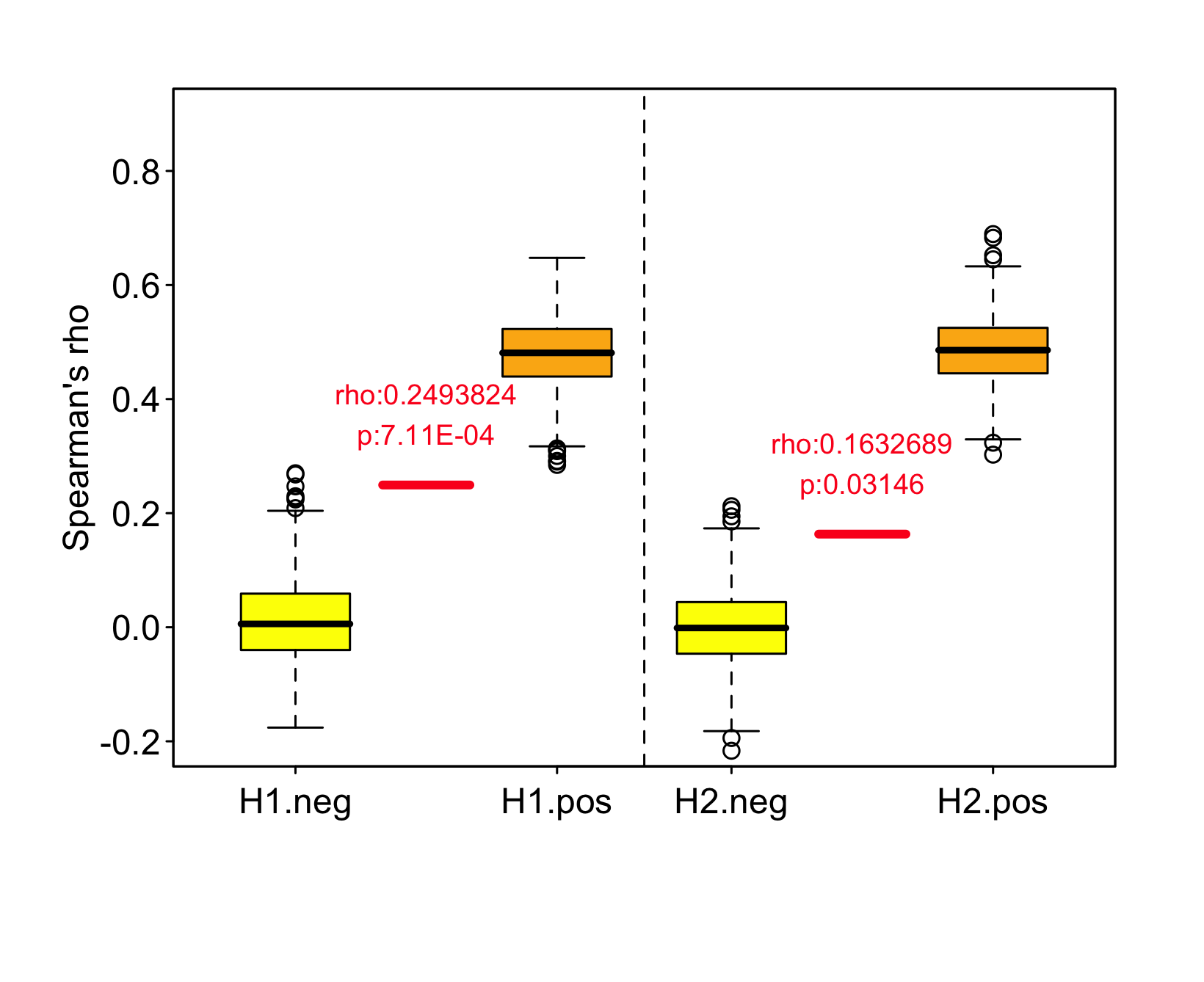


**Figure S9. Expression correlation between igHTR and the nearest protein-coding gene.** Red lines indicate the mean Spearman correlation *rho* between expression of igHTR located within 10 kb of the 3’-UTR of the nearest gene and expression of the nearest gene in human sample 1 (H1) and human sample 2 (H2). The red numbers indicate the exact *rho-* and *p*-values of the correlation. Positive control (orange) shows the distribution of mean correlation coefficients found between expression levels of the 3’-most exonic HTR and the rest of the gene estimated in the same number of pairs as the number of igHTR/gene pairs tested, randomly sampled from expressed genes 1,000 times. Negative control (yellow) shows the distribution of mean correlation coefficients found between expression levels in the same number of randomly composed pairs of igHTR and expressed genes as the number of clustering igHTR/gene pairs tested, randomly sampled 1,000 times. The *q*-values of observed igHTR/gene correlations based on the negative control distributions equal 0.002 for H1 and 0.005 for H2.
