# Supplementary material for: Intergenic and Repeat Transcription in Human, Chimpanzee and Macaque Brains Measured by RNA-Seq
Source: PLoS Comput Biol. 2010 Jul 1;6(7):e1000843. doi: 10.1371/journal.pcbi.1000843 (PMC2895644; doi:10.1371/journal.pcbi.1000843)
Supplement: Figure S10 — Number of igHTR/gene 3 prime end connections verified by at least one EST sequence (0.15 MB DOC) [file pcbi.1000843.s010.doc]

**Figure S10**

**
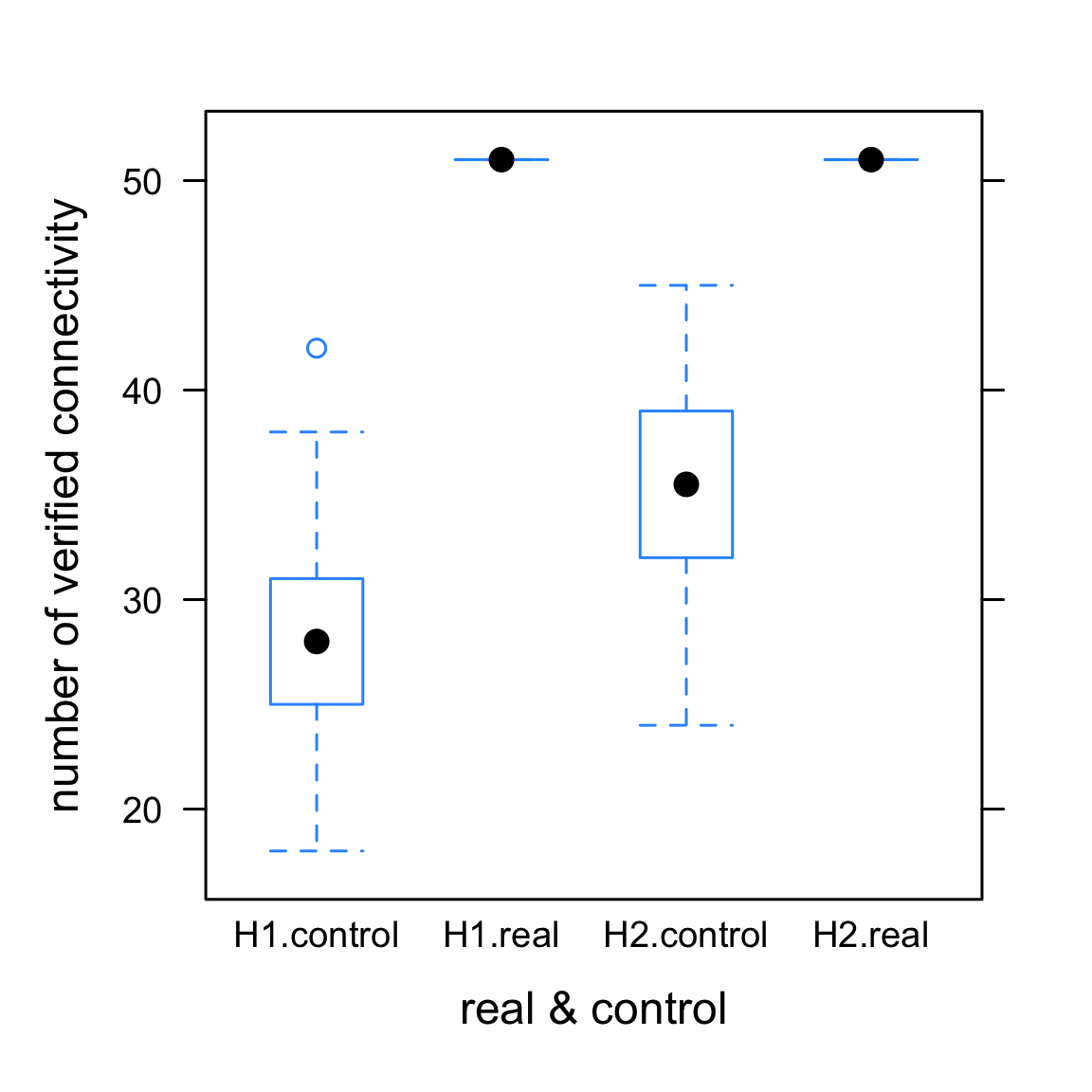
**

**Figure S10. Number of igHTR/gene 3’-end connections verified by at least one EST sequence.** The lines represent the number of EST-verified connections found between igHTR located within 10 kb of the 3’-UTR of the nearest gene in human sample 1 (H1) and human sample 2 (H2). All considered genes are expressed in our dataset and are represented by at least one exonic HTR. EST sequences originate from human brain EST libraries (LID_10197, LID_10279, LID_10284, LID_110, LID_111, LID_12525, LID_12527, LID_13046, LID_13053, LID_1318, LID_1331, LID_13711, LID_13865, LID_14181, LID_14271, LID_1446, LID_14591, LID_14592, LID_1535, LID_16376, LID_16378, LID_16380, LID_16382, LID_16383, LID_16384, LID_16390, LID_16394, LID_16411, LID_16437, LID_1703, LID_17380, LID_17384, LID_1749, LID_1750, LID_17681, LID_17761, LID_17762, LID_18304, LID_18309, LID_18310, LID_18311, LID_18312, LID_18313, LID_18314, LID_18315, LID_18317, LID_18318, LID_18319, LID_18322, LID_18323, LID_18324, LID_18325, LID_18348, LID_18349, LID_18352, LID_18353, LID_18358, LID_18375, LID_18415, LID_18466, LID_18467, LID_18472, LID_18508, LID_186, LID_1918, LID_19375, LID_19377, LID_19471, LID_208, LID_21, LID_2297, LID_2389, LID_2506, LID_263, LID_299, LID_2, LID_300, LID_318, LID_390, LID_394, LID_4525, LID_513, LID_5375, LID_5949, LID_5980, LID_629, LID_6682, LID_6756, LID_6758, LID_6811, LID_6812, LID_6814, LID_688, LID_7138, LID_7139, LID_742, LID_769, LID_8215, LID_8221, LID_8569, LID_8570, LID_8607, LID_8619, LID_8850, LID_9445, LID_9528, LID_9721, LID_9725, LID_9727) downloaded from NCBI’s library browser (http://www.ncbi.nlm.nih.gov/UniGene/lbrowse2.cgi). The boxes represent the distributions of EST-verified connections between randomly chosen genes expressed in our dataset and intergenic regions located within 10kb of their 3’-UTR, equal in number to the actual tested igHTR/gene pairs, in 1,000 simulations.

Our background estimates for EST-igHTR connection are high. This, however, could in part be caused by the choice of the background: here we use all genes with no identified igHTR within 10 kb downstream from gene. In our study, however, many real 3’-UTR extensions can be missed. They could fall below our igHTR detection cutoff or they could be expressed in other brain regions/ontogenetic stages. Such 3’-UTR extensions could still have corresponding EST sequences, thus resulting in high background “EST-connection” rate in our study.
