# Supplementary material for: Intergenic and Repeat Transcription in Human, Chimpanzee and Macaque Brains Measured by RNA-Seq
Source: PLoS Comput Biol. 2010 Jul 1;6(7):e1000843. doi: 10.1371/journal.pcbi.1000843 (PMC2895644; doi:10.1371/journal.pcbi.1000843)
Supplement: Figure S11 — Number of conserved miRNA target sites in igHTR (0.10 MB DOC) [file pcbi.1000843.s011.doc]

**Figure S11**

**
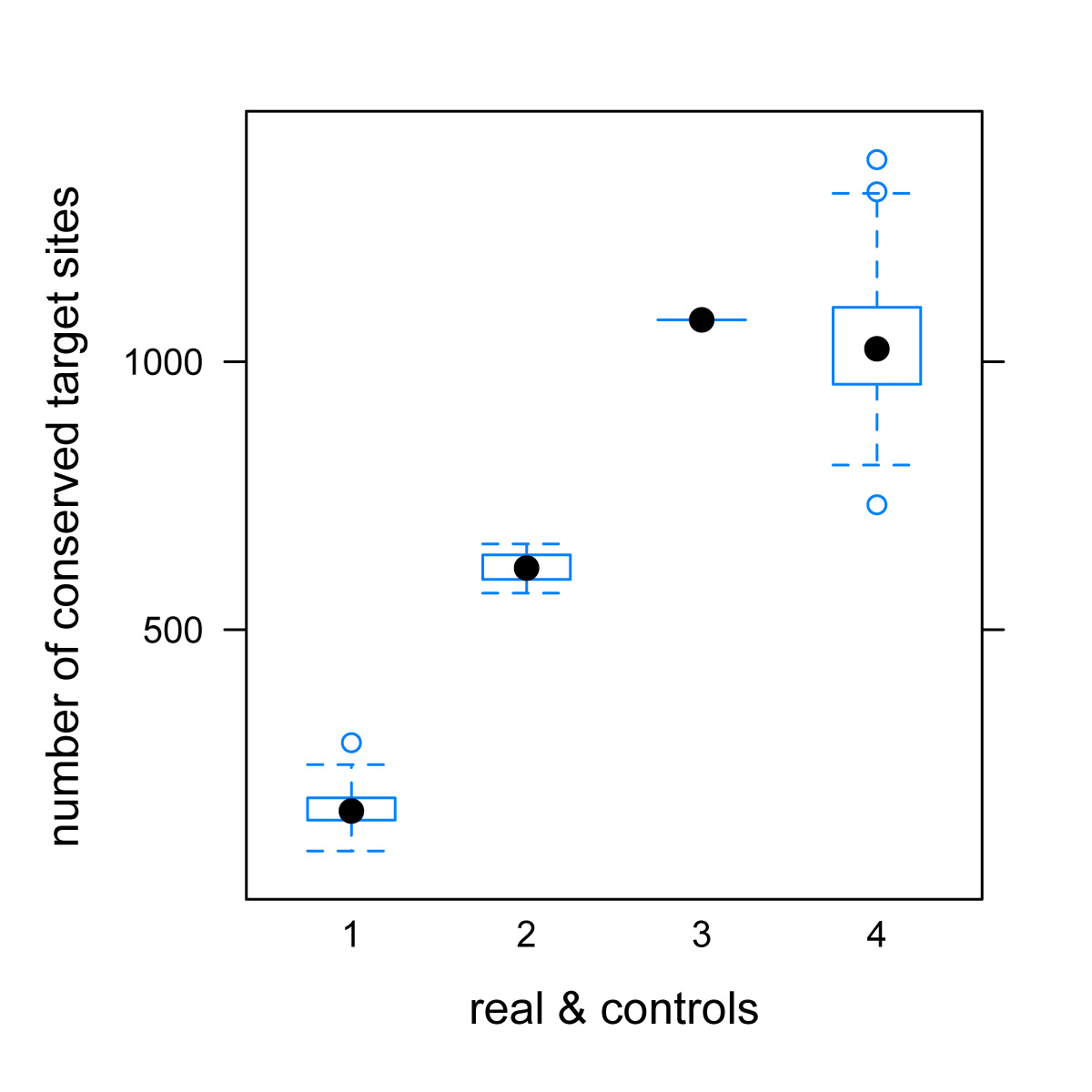
**

**Figure S11. Number of conserved miRNA target sites in igHTR.** The line (position 3) represents the number of miRNA binding sites identified by TargetScan in igHTR located within 10 kb of the 3’-UTR of the nearest gene using human-mouse alignment (downloaded from UCSC genome browser, http://genome.ucsc.edu/), according to the default TargetScan criteria. Negative control 1 (position 1): distribution of conserved miRNA binding sites within randomly sampled intergenic regions with the same number and the same length distribution as tested igHTR, calculated 100 times. Negative control 2 (position 2): the same as Negative control 1, except randomly sampled intergenic regions were additionally matched with tested igHTR for DNA sequnce conservation based on phastCons scores among 18 placental mammalian species. Positive control (position 4): distribution of conserved miRNA binding sites within randomly sampled 3’-UTR of annotated human genes with the same number and the same length distribution as tested igHTR, calculated 100 times. The boxes show variation of sampled number of conserved miRNA target sites and are drawn using function “bwplot” in R package “lattice” with no modification.
