# Supplementary material for: Intergenic and Repeat Transcription in Human, Chimpanzee and Macaque Brains Measured by RNA-Seq
Source: PLoS Comput Biol. 2010 Jul 1;6(7):e1000843. doi: 10.1371/journal.pcbi.1000843 (PMC2895644; doi:10.1371/journal.pcbi.1000843)
Supplement: Figure S12 — Codon Substitution Frequency (CSF) score in different types of regions (0.08 MB DOC) [file pcbi.1000843.s012.doc]

**Figure S12**

**
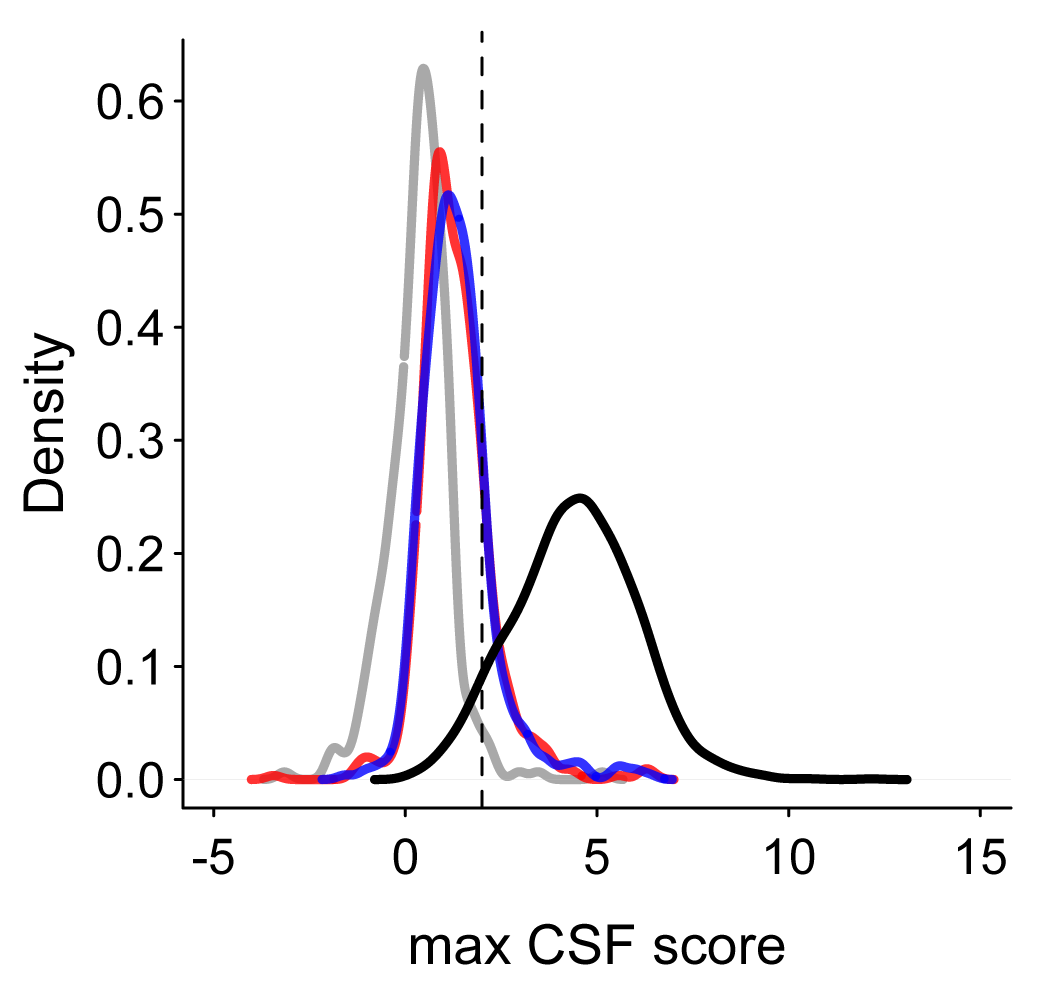
**

**Figure S12. Codon Substitution Frequency (CSF) score in different types of regions.** Distributions of CSF scores for igHTR (red – Human1, blue – Human2), annotated protein-coding genes (black), and annotated non-coding RNA (grey). Dashed line indicates the cutoff for discriminating coding of non-coding HTR at <5% false positive rate determined based on the distribution of CSF scores of annotated ncRNA (grey).
