# Supplementary material for: Intergenic and Repeat Transcription in Human, Chimpanzee and Macaque Brains Measured by RNA-Seq
Source: PLoS Comput Biol. 2010 Jul 1;6(7):e1000843. doi: 10.1371/journal.pcbi.1000843 (PMC2895644; doi:10.1371/journal.pcbi.1000843)
Supplement: Figure S13 — Overlap between igHTR and lincRNAs (0.10 MB DOC) [file pcbi.1000843.s013.doc]

**Figure S13**

**
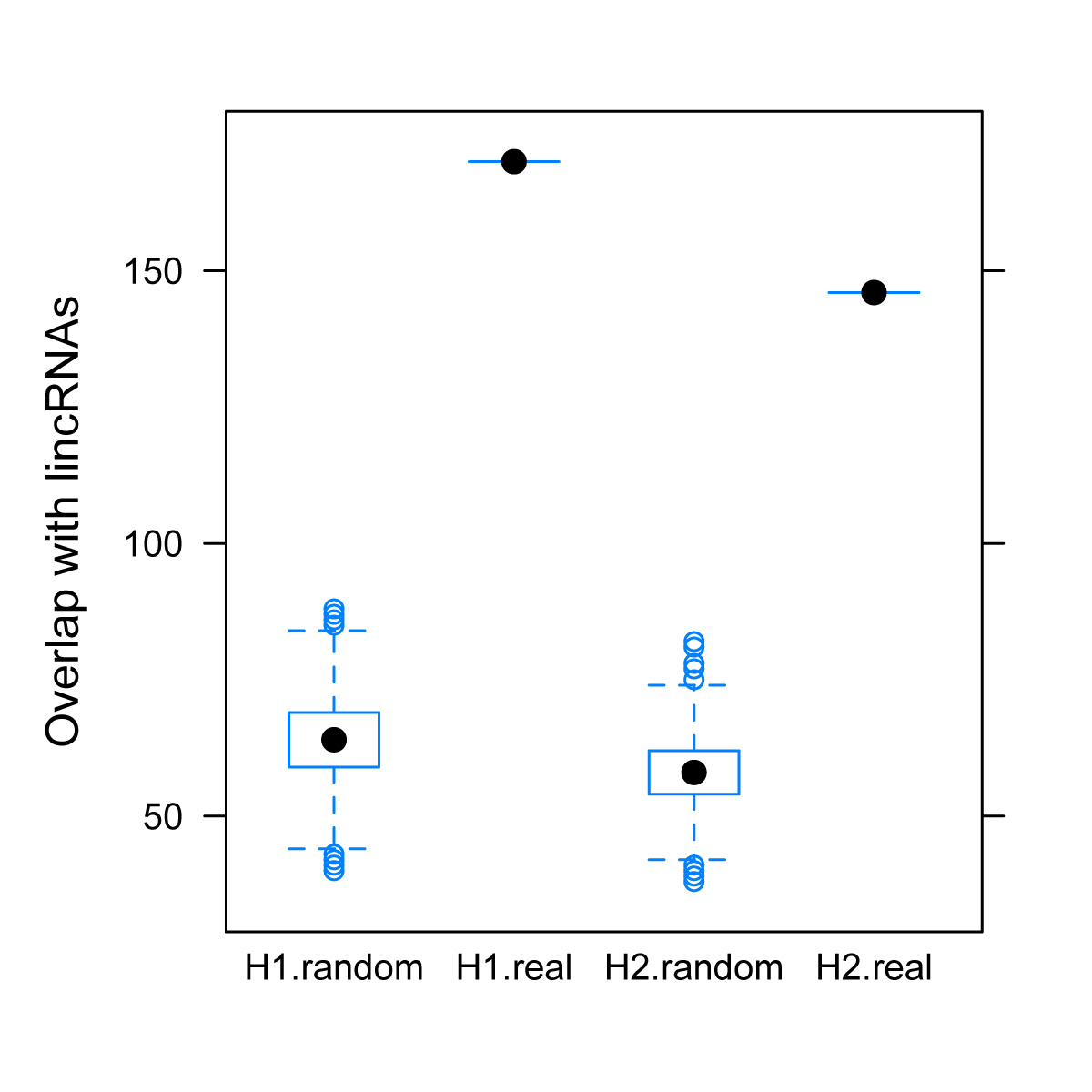
**

**Figure S13. Overlap between igHTR and lincRNAs.** The y-axis shows the number of igHTR overlapping with annotated lincRNA (see Methods for details). “H1”, “H2” indicate two human samples, “real” represents the observed overlap, while “random” stands for overlap based on randomly simulated igHTR in intergenic regions with the same number and length as the real igHTR 1,000 times. The boxes show variation of simulated overlap measurements and are drawn using function “bwplot” in R package “lattice” with no modification.
