# Supplementary material for: Intergenic and Repeat Transcription in Human, Chimpanzee and Macaque Brains Measured by RNA-Seq
Source: PLoS Comput Biol. 2010 Jul 1;6(7):e1000843. doi: 10.1371/journal.pcbi.1000843 (PMC2895644; doi:10.1371/journal.pcbi.1000843)
Supplement: Figure S14 — Overlap between igHTR and EvoFold predictions (0.10 MB DOC) [file pcbi.1000843.s014.doc]

**Figure S14**

**
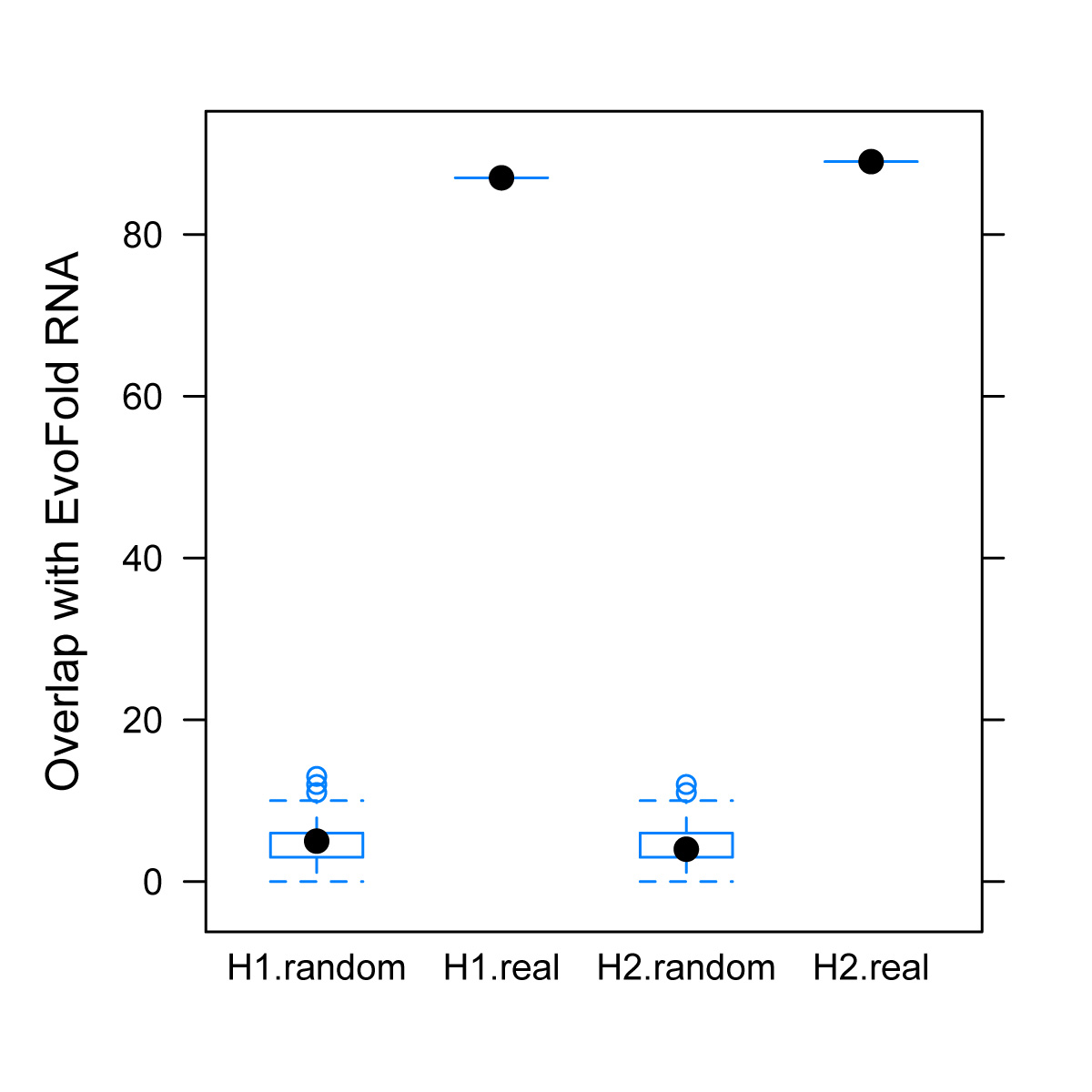
**

**Figure S14. Overlap between igHTR and EvoFold predictions.** The y-axis shows the number of igHTR overlapping with EvoFold ncRNA predictions. “H1”, “H2” indicate two human samples, “real” represents the observed overlap, while “random” stands for overlap based on randomly simulated igHTR in intergenic regions with the same number and length as the real igHTR 1,000 times. The boxes show variation of simulated overlap measurements and are drawn using function “bwplot” in R package “lattice” with no modification.
