# Supplementary material for: Intergenic and Repeat Transcription in Human, Chimpanzee and Macaque Brains Measured by RNA-Seq
Source: PLoS Comput Biol. 2010 Jul 1;6(7):e1000843. doi: 10.1371/journal.pcbi.1000843 (PMC2895644; doi:10.1371/journal.pcbi.1000843)
Supplement: Figure S15 — Gene expression trees based on different measures of gene expression divergence (0.16 MB DOC) [file pcbi.1000843.s015.doc]

**Figure S15**

**
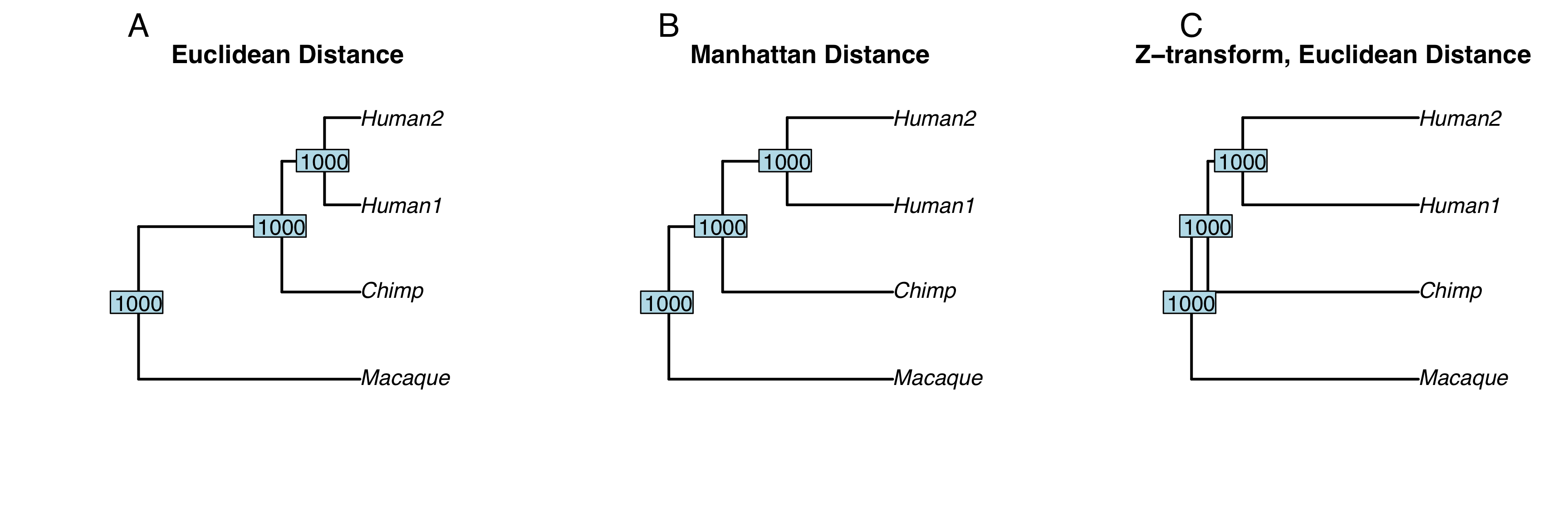
**

**Figure S15. Gene expression trees based on different measures of gene expression divergence.** Shown are UPGMA trees based on different expression level/expression divergence measurements. The expression divergence measurements used (see Methods for details): (**A**) Gene expression levels, Euclidean distance, (**B**) Gene expression levels, Manhattan distance, (**C**) Z-transformed expression levels, Euclidean distance. The numbers show node’s reproducibility based on 1,000 bootstraps over genes.
