# Supplementary material for: Intergenic and Repeat Transcription in Human, Chimpanzee and Macaque Brains Measured by RNA-Seq
Source: PLoS Comput Biol. 2010 Jul 1;6(7):e1000843. doi: 10.1371/journal.pcbi.1000843 (PMC2895644; doi:10.1371/journal.pcbi.1000843)
Supplement: Figure S16 — Gene expression divergence vs. evolutionary time (0.27 MB DOC) [file pcbi.1000843.s016.doc]

**Figure S16**

**
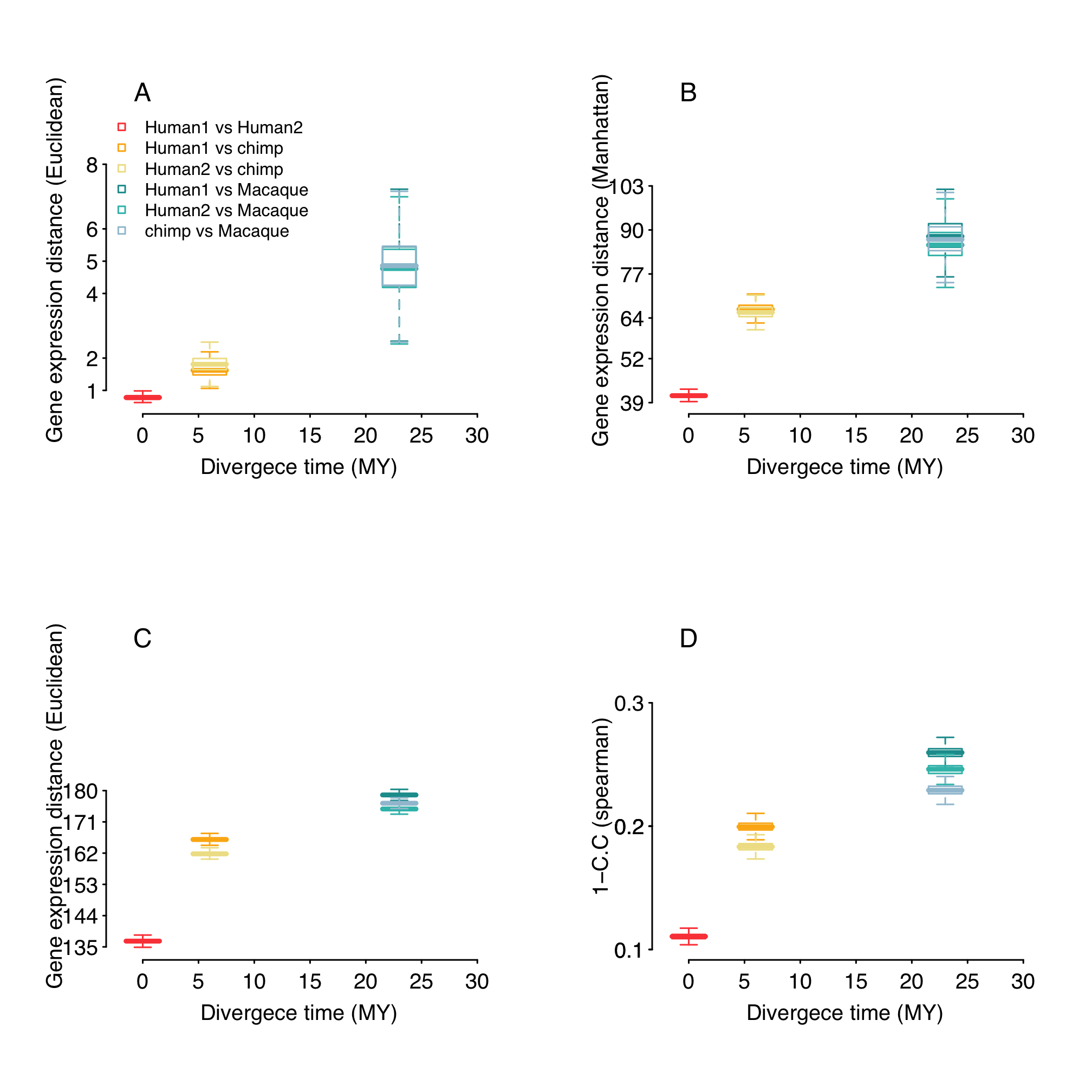
**

**Figure S16. Gene expression divergence *vs.* evolutionary time.** Shown are pairwise expression divergence estimates based on different expression level/expression divergence measurements (y-axis), plotted against time of species divergence in million years (MY) according to [32]. Human-human divergence was set to 0.1 MY. The expression divergence measurements used (see Methods for details): (**A**) Gene expression levels, Euclidean distance, (**B**) Gene expression levels, Manhattan distance, (**C**) Z-transformed expression levels, Euclidean distance, (**D**) 1-Spearman's correlation coefficient of genes expression levels. The boxplots (produced by “boxplot” function in R with default parameters) show variation of expression divergence estimates based on 1,000 bootstraps over genes.
