# Supplementary material for: Intergenic and Repeat Transcription in Human, Chimpanzee and Macaque Brains Measured by RNA-Seq
Source: PLoS Comput Biol. 2010 Jul 1;6(7):e1000843. doi: 10.1371/journal.pcbi.1000843 (PMC2895644; doi:10.1371/journal.pcbi.1000843)
Supplement: Figure S17 — Genomic annotation of expressed regions within HTR (0.21 MB DOC) [file pcbi.1000843.s017.doc]

**Figure S17**

**
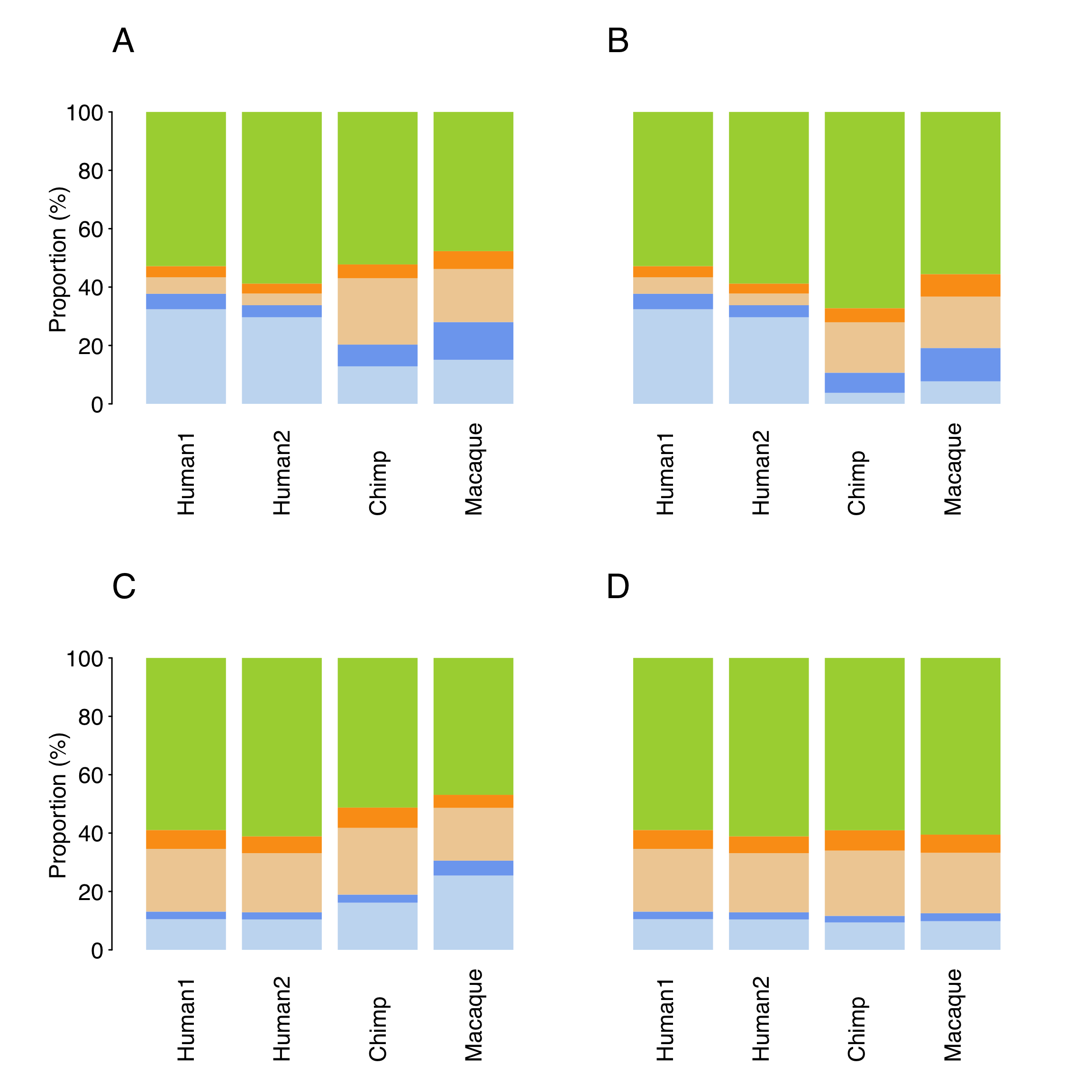
**

**Figure S17. Genomic annotation of expressed regions within HTR.** Each bar shows proportions of expressed nucleotides falling within HTR and annotated as exons (green), intronic repeats (orange), introns (light orange), intergenic repeats (blue), and intergenic regions (light blue). (**A**) HTR with species-specific expression, annotation according to the each species’ genome. (**B**) HTR with species-specific expression, regions were projected onto the human genome and annotated according to the human genome annotation. (**C**) All HTR, annotation according to the each species’ genome. (**D**) All HTR, regions were projected onto the human genome and annotated according to the human genome annotation. The exact proportions for each category are listed in the Table S9.
