# Supplementary material for: Intergenic and Repeat Transcription in Human, Chimpanzee and Macaque Brains Measured by RNA-Seq
Source: PLoS Comput Biol. 2010 Jul 1;6(7):e1000843. doi: 10.1371/journal.pcbi.1000843 (PMC2895644; doi:10.1371/journal.pcbi.1000843)
Supplement: Figure S18 — Genomic annotation of expressed regions within GW (0.21 MB DOC) [file pcbi.1000843.s018.doc]

**Figure S18**

**
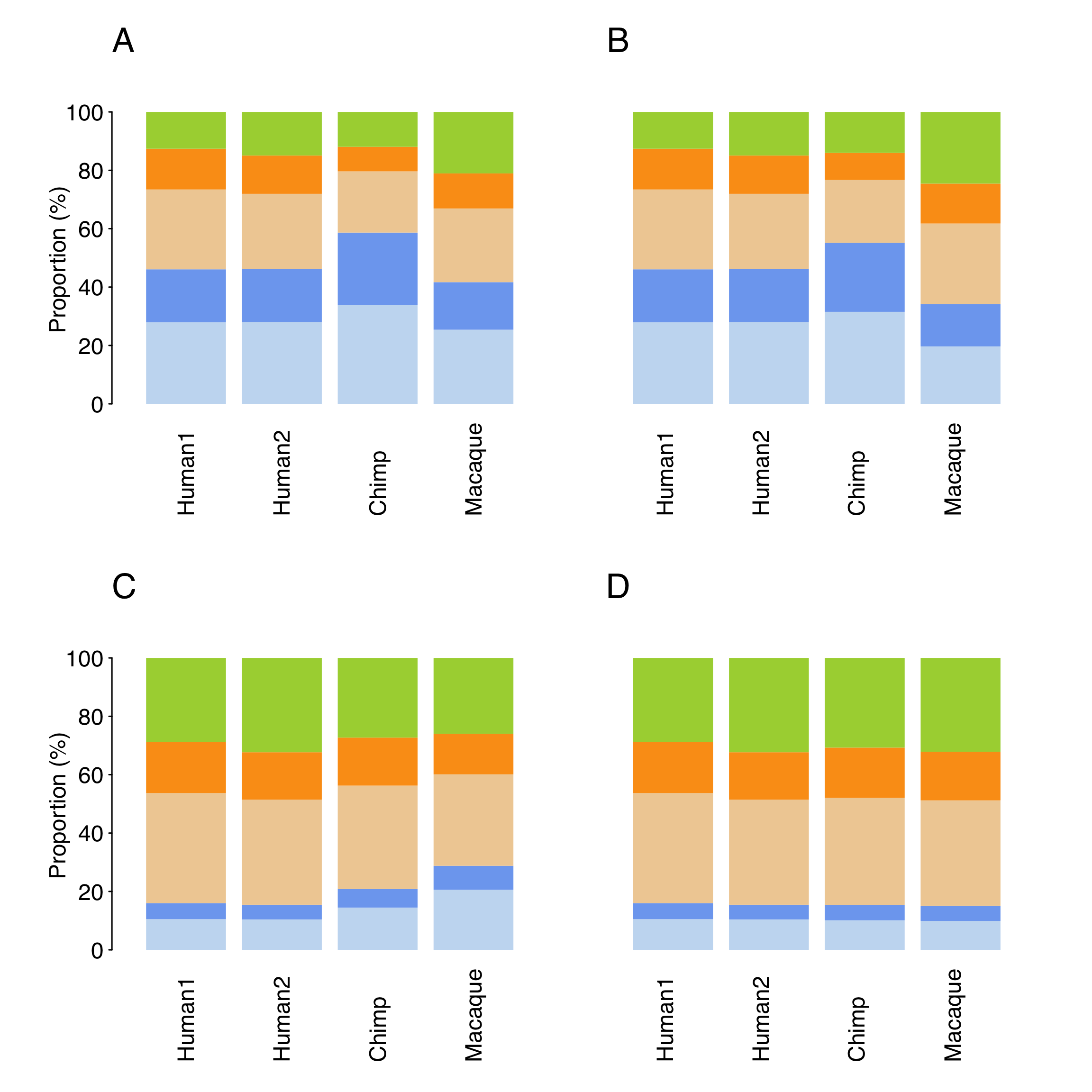
**

**Figure S18. Genomic annotation of expressed regions within GW.** Each bar shows proportions of expressed nucleotides falling within GW and annotated as exons (green), intronic repeats (orange), introns (light orange), intergenic repeats (blue), and intergenic regions (light blue). (**A**) GW with species-specific expression, annotation according to the each species’ genome. (**B**) GW with species-specific expression, regions were projected onto the human genome and annotated according to the human genome annotation. (**C**) All GW, annotation according to the each species’ genome. (**D**) All GW, regions were projected onto the human genome and annotated according to the human genome annotation. The exact proportions for each category are listed in the Table S11.
